# Chimeric spike mRNA vaccines protect against Sarbecovirus challenge in mice

**DOI:** 10.1126/science.abi4506

**Published:** 2021-08-27

**Authors:** David R. Martinez, Alexandra Schäfer, Sarah R. Leist, Gabriela De la Cruz, Ande West, Elena N. Atochina-Vasserman, Lisa C. Lindesmith, Norbert Pardi, Robert Parks, Maggie Barr, Dapeng Li, Boyd Yount, Kevin O. Saunders, Drew Weissman, Barton F. Haynes, Stephanie A. Montgomery, Ralph S. Baric

**Affiliations:** 1 Department of Epidemiology, University of North Carolina at Chapel Hill, Chapel Hill, NC, USA.; 2 Lineberger Comprehensive Cancer Center, University of North Carolina School of Medicine, Chapel Hill, NC, USA.; 3 Infectious Disease Division, Department of Medicine, Perelman School of Medicine, University of Pennsylvania Perelman School of Medicine, Philadelphia, PA, USA.; 4 Duke Human Vaccine Institute, Duke University School of Medicine, Durham, NC, USA.; 5 Department of Laboratory Medicine and Pathology, University of North Carolina School of Medicine, Chapel Hill, NC, USA.

## Abstract

The emergence of severe acute respiratory syndrome coronavirus (SARS-CoV) in 2003 and SARS-CoV-2 in 2019 highlights the need to develop universal vaccination strategies against the broader *Sarbecovirus* subgenus. Using chimeric spike designs, we demonstrate protection against challenge from SARS-CoV, SARS-CoV-2, SARS-CoV-2 B.1.351, bat CoV (Bt-CoV) RsSHC014, and a heterologous Bt-CoV WIV-1 in vulnerable aged mice. Chimeric spike messenger RNAs (mRNAs) induced high levels of broadly protective neutralizing antibodies against high-risk Sarbecoviruses. By contrast, SARS-CoV-2 mRNA vaccination not only showed a marked reduction in neutralizing titers against heterologous Sarbecoviruses, but SARS-CoV and WIV-1 challenge in mice resulted in breakthrough infections. Chimeric spike mRNA vaccines efficiently neutralized D614G, mink cluster five, and the UK B.1.1.7 and South African B.1.351 variants of concern. Thus, multiplexed-chimeric spikes can prevent SARS-like zoonotic coronavirus infections with pandemic potential.

## A broad defense against SARS-like viruses

Severe acute respiratory syndrome coronavirus 2 (SARS-CoV-2) is the third coronavirus that has emerged as a serious human pathogen in the past 20 years. Treatment strategies that are broadly protective against current and future SARS-like coronaviruses are needed. Martinez *et al*. take on this challenge by developing vaccines based on chimeras of the viral spike protein. The messenger RNA vaccines encode spike proteins composed of domain modules from epidemic and pandemic coronaviruses, as well as bat coronaviruses with the potential to cross to humans. In aged mice vulnerable to infection, the chimeric vaccines protected against challenge from SARS-CoV, SARS-CoV-2 and tested variants of concern, and zoonotic coronaviruses with pandemic potential. —VV

**Figure:**
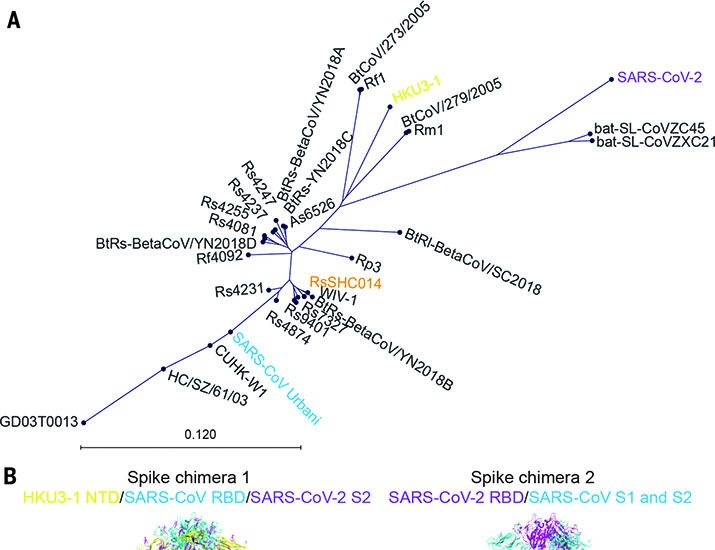

## Teaser

mRNA vaccines that express chimeric spike protein protect mice against epidemic, zoonotic, and pandemic SARS-like viruses.

A novel severe acute respiratory syndrome coronavirus (SARS-CoV) emerged in 2003 and caused more than 8000 infections and ~800 deaths worldwide (*1*). In 2012, the Middle East respiratory syndrome coronavirus (MERS-CoV) emerged in Saudi Arabia (*2*), with multiple outbreaks that have resulted in at least ~2600 cases and 900 deaths (*3*). In December 2019, another novel human SARS-like virus from the genus *Betacoronavirus* and subgenus *Sarbecovirus* emerged in Wuhan China, designated SARS-CoV-2, causing the ongoing COVID-19 pandemic (*4*, *5*).

Bats are known reservoirs of SARS-like coronaviruses (CoVs) and harbor high-risk “preemergent” SARS-like variant strains, such as WIV-1-CoV and RsSHC014-CoV, which are able to use human ACE2 (angiotensin-converting enzyme 2) receptors for entry, replicate efficiently in human primary airway epithelial cells, and may escape existing countermeasures (*6*, *7*). Given the high pandemic potential of zoonotic and epidemic Sarbecoviruses (*8*), the development of countermeasures such as broadly effective vaccines, antibodies, and drugs is a global health priority (*9*–*11*).

Sarbecovirus spike proteins have immunogenic domains: the receptor binding domain (RBD), the N-terminal domain (NTD), and the subunit 2 (S2) (*12*, *13*). RBD, NTD, and to a lesser extent S2 are targets for potent neutralizing and non-neutralizing antibodies elicited to SARS-CoV-2 and MERS-CoV spike (*12*, *14*–*19*). Passive immunization with SARS-CoV-2 NTD-specific antibodies protect naïve mice from challenge, demonstrating that the NTD is a target of protective immunity (*12*, *19*, *20*). However, it remains unclear whether vaccine-elicited neutralizing antibodies can protect against in vivo challenge with heterologous epidemic and bat coronaviruses. We generated nucleoside-modified mRNA-lipid nanoparticle (LNP) vaccines expressing chimeric spikes that contain admixtures of different RBD, NTD, and S2 modular domains from zoonotic, epidemic, and pandemic CoVs and examined their efficacy against homologous and heterologous Sarbecovirus challenge in aged mice.

## Results

### Design and expression of chimeric spike constructs to cover pandemic and zoonotic SARS-related coronaviruses

**Fig. 1.**
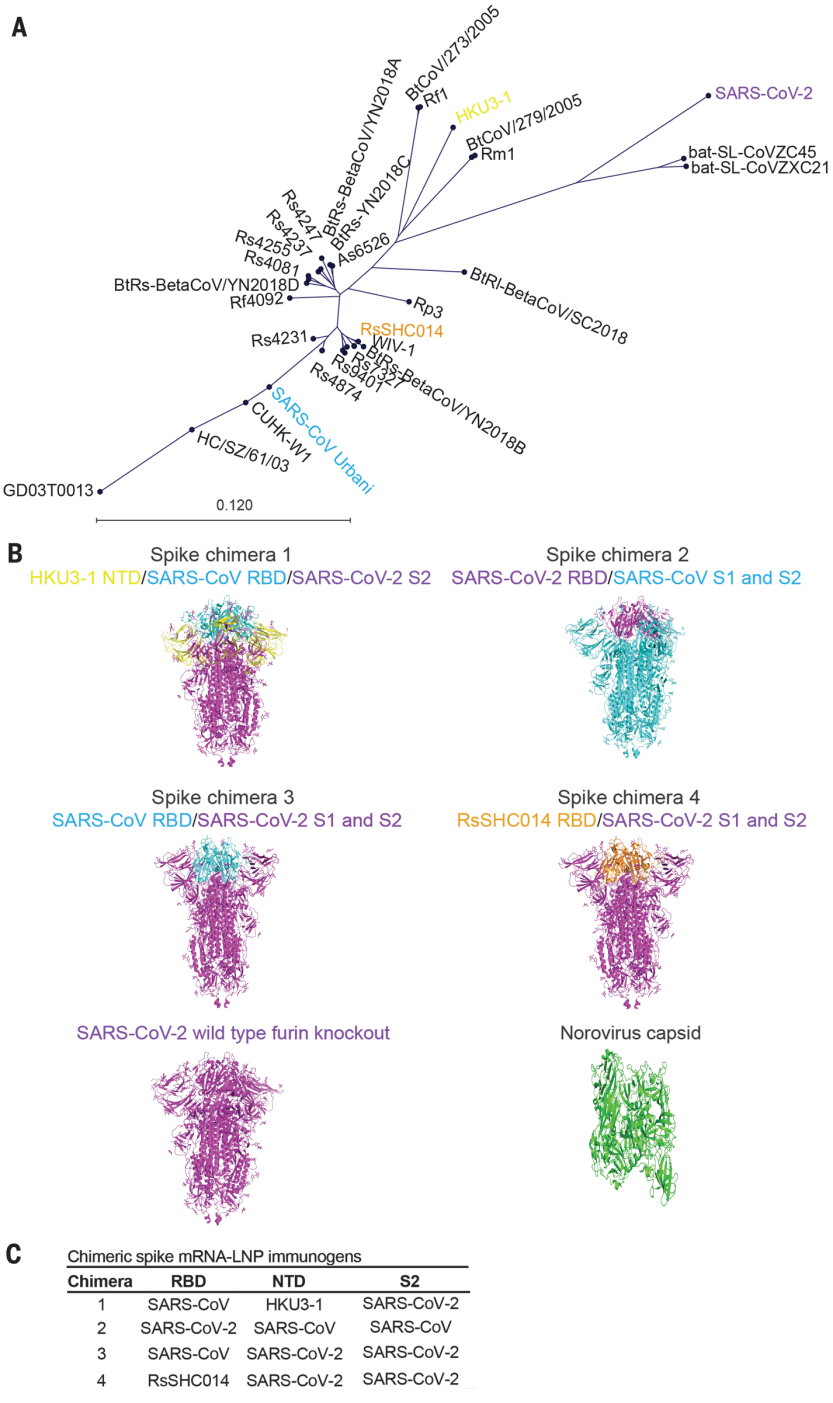
Genetic design of chimeric Sarbecovirus spike vaccines. (**A**) Genetic diversity of pandemic and bat zoonotic coronaviruses. HKU3-1 is shown in yellow, SARS-CoV is shown in light blue, RsSHC014 is shown in orange, and SARS-CoV-2 is shown in purple. (**B**) Spike chimera 1 includes the NTD from HKU3-1, the RBD from SARS-CoV, and the rest of the spike from SARS-CoV-2. Spike chimera 2 includes the RBD from SARS-CoV-2 and the NTD and S2 from SARS-CoV. Spike chimera 3 includes the RBD from SARS-CoV and the NTD and S2 SARS-CoV-2. Spike chimera 4 includes the RBD from RsSHC014 and the rest of the spike from SARS-CoV-2. SARS-CoV-2 furin KO spike vaccine and is the norovirus capsid vaccine. (**C**) Table summary of chimeric spike constructs.

Sarbecoviruses exhibit considerable genetic diversity (Fig. 1A), and SARS-like bat CoVs (Bt-CoVs) are recognized threats to human health (*6*, *8*). Because potent neutralizing antibody epitopes exist in each of the modular structures on CoV spikes (*21*), we hypothesized that chimeric spikes that encode NTD, RBD, and S2 domains into “bivalent” and “trivalent” vaccine immunogens have the potential to elicit broad protective antibody responses against clades I to III Sarbecoviruses. We designed four sets of chimeric spikes. Chimera 1 included the NTD from clade II Bt-CoV Hong Kong University 3-1 (HKU3-1), the clade I SARS-CoV RBD, and the clade III SARS-CoV-2 S2 (Fig. 1B). Chimera 2 included SARS-CoV-2 RBD and SARS-CoV NTD and S2 domains (*11*). Chimera 3 included the SARS-CoV RBD and SARS-CoV-2 NTD and S2, whereas chimera 4 included the RsSHC014 RBD and SARS-CoV-2 NTD and S2. We also generated a monovalent SARS-CoV-2 spike furin knockout (KO) vaccine, partially phenocopying the Moderna and Pfizer mRNA vaccines in human use, and a negative control norovirus GII capsid vaccine (Fig. 1, B and C). We generated these chimeric spikes and control spikes as lipid nanoparticle-encapsulated, nucleoside-modified mRNA vaccines with LNP adjuvants (mRNA-LNP), as described previously (*22*). This mRNA LNP stimulates robust T follicular helper cell activity, germinal center B cell responses, durable long-lived plasma cells, and memory B cell responses (*23*, *24*). We verified their chimeric spike expression in human embryonic kidney (HEK) cells (fig. S1B). To confirm that scrambled coronavirus spikes are biologically functional, we also designed and recovered several high-titer recombinant live viruses of RsSHC014/SARS-CoV-2 NTD, RBD, and S2 domain chimeras that included deletions in nonessential, accessory open reading frame 7 (ORF7) and ORF8 that encoded nanoluciferase (fig. S1C). SARS-CoV-2 ORF7 and -8 antagonize innate immune signaling pathways (*25*, *26*), and deletions in these ORFs are associated with attenuated disease in humans (*27*, *28*).

### Immunogenicity of mRNAs expressing chimeric spike constructs against coronaviruses

We next sought to determine whether simultaneous immunization with mRNA-LNP expressing the chimeric spikes of diverse Sarbecoviruses was a feasible strategy to elicit broad binding and neutralizing antibodies. We immunized aged mice with the chimeric spikes formulated to induce cross-reactive responses against multiple divergent clades I to III Sarbecoviruses, a SARS-CoV-2 furin KO spike, and a GII.4 norovirus capsid negative control. Group 1 was primed and boosted with chimeric spikes 1, 2, 3, and 4 (fig. S1A). Group 2 was primed with chimeric spikes 1 and 2 and boosted with chimeric spikes 3 and 4 (fig. S1A). Group 3 was primed and boosted with chimeric spike 4 (fig. S1A). Group 4 was primed and boosted with the monovalent SARS-CoV-2 furin KO spike (fig. S1A). Last, group 5 was primed and boosted with a norovirus capsid GII.4 Sydney 2011 strain (fig. S1A). We then examined the binding antibody responses by means of enzyme-linked immunosorbent assay (ELISA) against a diverse panel of CoV spike proteins that included epidemic, pandemic, and zoonotic coronaviruses.

**Fig. 2.**
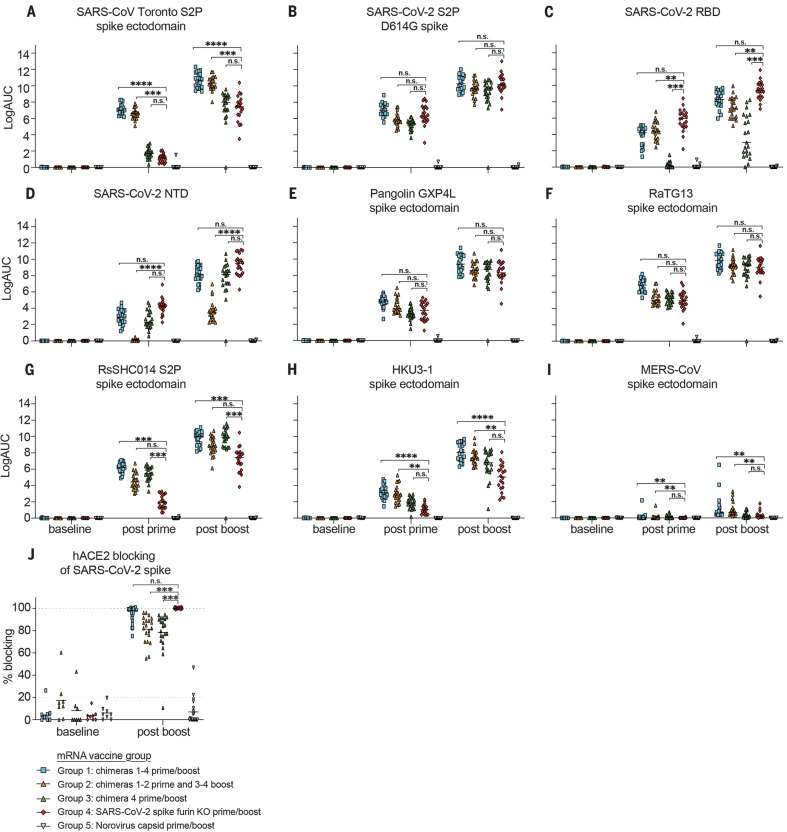
Human and animal coronavirus spike binding and hACE2-blocking responses in chimeric and monovalent SARS-CoV-2 spike-vaccinated mice. Serum antibody ELISA binding responses were measured in the five different vaccination groups. Before immunization, after prime, and after boost binding responses were evaluated against Sarbecoviruses, MERS-CoV, and common-cold CoV antigens including (**A**) SARS-CoV Toronto Canada (Tor2) S2P, (**B**) SARS-CoV-2 S2P D614G, (**C**) SARS-CoV-2 RBD, (**D**) SARS-CoV-2 NTD, (**E**) Pangolin GXP4L spike, (**F**) RaTG13 spike, (**G**) RsSHC014 S2P spike, (**H**) HKU3-1 spike, (**I**) MERS-CoV spike, and (**J**) hACE2 blocking responses against SARS-CoV-2 spike in the distinct immunization groups. Blue squares indicate mice from group 1, orange triangles indicate mice from group 2, green triangles indicate mice from group 3, red rhombuses indicate mice from group 4, and upside-down triangles indicate mice from group 5. Statistical significance for the binding and blocking responses is reported from a Kruskal-Wallis test after Dunnett’s multiple comparison correction. *P < 0.05, **P < 0.01, ***P < 0.001, and ****P < 0.0001.

Mice in groups 1 and 2 generated the highest-magnitude responses to SARS-CoV Toronto Canada isolate (Tor2), RsSHC014, and HKU3-1 spike as compared with group 4 (Fig. 2, A, G, and H). Whereas mice in group 2 generated lower-magnitude binding responses to both SARS-CoV-2 RBD (Fig. 2C) and SARS-CoV-2 NTD (Fig. 2D), mice in group 1 generated similar-magnitude binding antibodies to SARS-CoV-2 D614G (in which aspartic acid at position 614 is replaced with glycine) as compared with that of mice immunized with the SARS-CoV-2 furin KO spike mRNA-LNP (Fig. 2B). Mice in groups 1 and 2 generated similar-magnitude binding antibody responses against SARS-CoV-2 D614G, Pangolin GXP4L, and RaTG13 spikes (Fig. 2, B, E, and F) compared with those of mice from group 4. Mice in group 1 and group 4 elicited high-magnitude levels of hACE2 blocking responses, as compared with those of groups 2 and 3 (Fig. 2J). Because binding antibody responses after boost mirrored the trend of the after-prime responses, it is likely that the second dose is boosting immunity to the vaccine antigens in the prime (Fig. 2). Last, we did not observe cross-binding antibodies against common-cold CoV spike antigens from HCoV-HKU1, HCoV-NL63, and HCoV-229E in most of the vaccine groups (fig. S2, A to D), but we did observe low binding levels against more distant group 2C MERS-CoV (Fig. 2I) and other Betacoronaviruses such as group 2A HCoV-OC43 in vaccine groups 1 and 2 (fig. S2B). These results suggest that chimeric spike vaccines elicit broader and higher-magnitude binding responses against pandemic and bat SARS-like viruses as compared with those of monovalent SARS-CoV-2 spike vaccines.

### Neutralizing antibody responses against live Sarbecoviruses and variants of concern

**Fig. 3.**
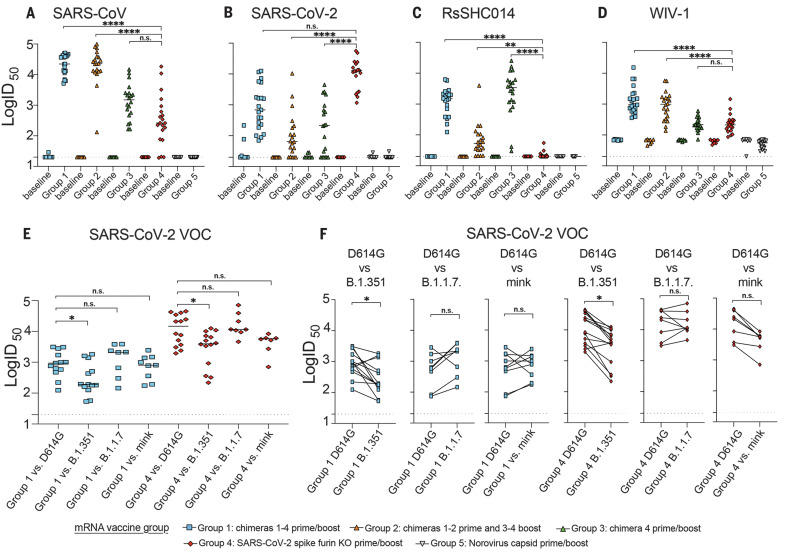
Live Sarbecovirus neutralizing antibody responses in vaccinated mice. Neutralizing antibody responses in mice from the five different vaccination groups were measured by using nanoluciferase-expressing recombinant viruses. (**A**) SARS-CoV neutralizing antibody responses from baseline and after boost in the distinct vaccine groups. (**B**) SARS-CoV-2 neutralizing antibody responses from baseline and after boost. (**C**) RsSHC014 neutralizing antibody responses from baseline and after boost. (**D**) WIV-1 neutralizing antibody responses from baseline and after boost. (**E**) The neutralization activity in groups 1 and 4 against SARS-CoV-2 D614G, South African B.1.351, UK B.1.1.7, and mink cluster 5 variant. (**F**) Neutralization comparison of SARS-CoV-2 D614G versus South African B.1.351, versus UK B.1.1.7, and versus mink cluster 5 variant. Statistical significance for the live-virus neutralizing antibody responses is reported from a Kruskal-Wallis test after Dunnett’s multiple comparison correction. *P < 0.05, **P < 0.01, ***P < 0.001, and ****P < 0.0001.

We then examined the neutralizing antibody responses against SARS-CoV, Bt-CoV RsSHC014, Bt-CoV WIV-1, and SARS-CoV-2 including variants of concern using live viruses as previously described (Fig. 3, A to D) (*17*). Group 4 SARS-CoV-2 S mRNA–vaccinated animals mounted a robust response against SARS-CoV-2; however, responses against SARS-CoV, RsSHC014, and WIV-1 were 18-, >300- or 116-fold decreased, respectively (Fig. 3, A to D, and fig. S3, G and H). By contrast, aged mice in group 2 showed a 42- and twofold increase in neutralizing titer against SARS-CoV and WIV1 and less than onefold decrease against RsSHC014 relative to SARS-CoV-2 neutralizing titers (Fig. 3, A to D, and fig. S3, C and D). Mice in group 3 elicited thee- and sevenfold increased neutralizing titers against SARS-CoV and RsSHC014 yet showed a threefold decrease in WIV-1 neutralizing titers relative to SARS-CoV-2 (Fig. 3, A to D, and fig. S3, E and F). Last, mice in group 1 generated the most balanced and highest neutralizing titers, which were 13- and 1.2-fold increased against SARS-CoV and WIV-1 and less than onefold decreased against RsSHC014 relative to the SARS-CoV-2 neutralizing titers (Fig. 3, A to D, and fig. S3, A and B). The serum of mice from groups 1 and 4 neutralized the dominant D614G variant with similar potency as that of the wild-type D614 nonpredominant variant, and both groups had similar neutralizing antibody responses against the UK B.1.1.7 and the mink cluster 5 variant as compared with the D614G variant (Fig. 3, E and F). Despite the significant but small reduction in neutralizing activity against the B.1.351 variant of concern (VOC), we did not observe a complete ablation in neutralizing activity in either group. Mice from groups 1 and 2 elicited lower binding and neutralizing responses to SARS-CoV-2 as compared with those of group 4, perhaps reflecting a decreased amount of mRNA vaccine incorporated into multiplexed formulations; the monovalent vaccines may drive a more focused B cell response to SARS-CoV-2, whereas chimeric spike antigens lead to more breadth against distant Sarbecoviruses. Thus, both monovalent SARS-CoV-2 vaccines and multiplexed chimeric spikes elicit neutralizing antibodies against newly emerged SARS-CoV-2 variants, and multiplexed chimeric spike vaccines outperform the monovalent SARS-CoV-2 vaccines in terms of breadth against multiclade Sarbecoviruses.

### In vivo protection against heterologous Sarbecovirus challenge

**Fig. 4.**
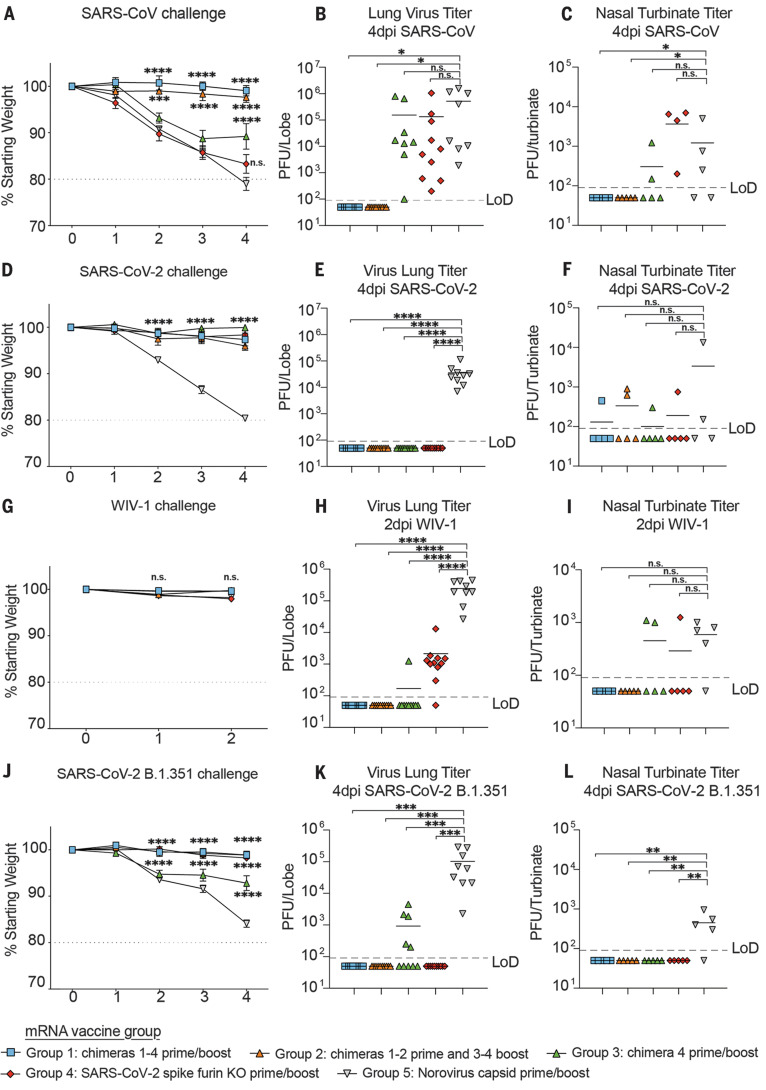
In vivo protection against Sarbecovirus challenge after mRNA-LNP vaccination. (**A**) Percent starting weight from the different vaccine groups of mice challenged with SARS-CoV MA15. (**B**) SARS-CoV MA15 lung viral titers in mice from the distinct vaccine groups. (**C**) SARS-CoV MA15 nasal turbinate titers. (**D**) Percent starting weight from the different vaccine groups of mice challenged with SARS-CoV-2 MA10. (**E**) SARS-CoV-2 MA10 lung viral titers in mice from the distinct vaccine groups. (**F**) SARS-CoV-2 MA10 nasal turbinate titers. (**G**) Percent starting weight from the different vaccine groups of mice challenged with WIV-1. (**H**) WIV-1 lung viral titers in mice from the distinct vaccine groups. (**I**) WIV-1 nasal turbinate titers. (**J**) Percent starting weight from the different vaccine groups of mice challenged with SARS-CoV-2 B.1.351. (**K**) SARS-CoV-2 B.1.351 lung viral titers in mice from the distinct vaccine groups. (**L**) SARS-CoV-2 B.1.351 nasal turbinate titers. The vaccines used in the different groups are denoted at bottom. Statistical significance for weight loss is reported from a two-way analysis of variance (ANOVA) after Dunnett’s multiple comparison correction. For lung and nasal turbinate titers, statistical significance is reported from a one-way ANOVA after Tukey’s multiple comparison correction. *P < 0.05, **P < 0.01, ***P < 0.001, and ****P < 0.0001.

To assess the ability of the mRNA-LNP vaccines to mediate protection against previously epidemic SARS-CoV, pandemic SARS-CoV-2, and Bt-CoVs, we challenged the different groups and observed the mice for signs of clinical disease. Mice from group 1 or group 2 were completely protected from weight loss and lower- and upper-airway virus replication as measured with infectious virus plaque assays after 2003 SARS-CoV mouse-adapted (MA15) challenge (Fig. 4, A, B, and C). Similarly, these two vaccine groups were also protected against SARS-CoV-2 mouse-adapted (MA10) challenge. By contrast, group 3 showed some protection against SARS-CoV MA15–induced weight loss but not against viral replication in the lung or nasal turbinates. Group 3 was fully protected against SARS-CoV-2 MA10 challenge. By contrast, group 5 vaccinated mice developed severe disease, including mortality in both SARS-CoV MA15 and SARS-CoV-2 MA10 infections (fig. S5, B and C). Monovalent SARS-CoV-2 mRNA vaccines were highly efficacious against SARS-CoV-2 MA10 challenge but failed to protect against SARS-CoV MA15–induced weight loss and replication in the lower and upper respiratory tract (Fig. 4, A, B, and C), suggesting that SARS-CoV-2 mRNA-LNP vaccines are not likely to protect against future SARS-CoV emergence events. Mice from groups 1 to 4 were completely protected from weight loss and lower airway SARS-CoV-2 MA10 replication (Fig. 4, D, E, and F). Using both a Bt-CoV RsSHC014 full-length virus and a more virulent RsSHC014-MA15 chimera in mice (*6*), we also demonstrated protection in groups 1 to 3 against RsSHC014 replication in the lung and nasal turbinates (fig. S4) but not in mice that received the SARS-CoV-2 mRNA vaccine. Group 5 control mice challenged with RsSHC014-MA15 developed disease, including mortality (fig. S5D). Group 3 mice, which received a SARS-CoV-2 NTD/RsSHC014 RBD/SARS-CoV-2 S2, were fully protected against both SARS-CoV-2 and RsSHC014 challenge, whereas group 4 mice were not, demonstrating that a single NTD and RBD chimeric spike can protect against more than one virus compared with a monovalent spike.

We then performed a heterologous challenge experiment with the bat preemergent WIV-1-CoV (*7*). Mice from groups 1 and 2 were fully protected against heterologous WIV-1 challenge, whereas mice that received the SARS-CoV-2 mRNA vaccine had breakthrough replication in the lung (Fig. 4, G, H, and I). We also challenged with a virulent form of SARS-CoV-2 VOC B.1.351, which contains deletions in the NTD and mutations in the RBD, and observed full protection in vaccine groups 1, 2, and 4 compared with that in controls, whereas breakthrough replication was observed in group 3, further indicating the importance of the NTD in vaccine-mediated protection (Fig. 4, J, K, and L). The reduced protection against the B.1.351 variant containing NTD deletions indicates that the NTD is a clear target of protective immunity and that its inclusion in vaccination strategies, as opposed to RBD-alone vaccines, may be required to achieve full protection. Moreover, the SARS-CoV-2 mRNA vaccine protected against SARS-CoV-2 B.1.351 challenge in aged mice despite a reduction in the neutralizing activity against this VOC.

### Lung pathology and cytokines in mRNA-LNP–vaccinated mice challenged with epidemic and pandemic coronaviruses

**Fig. 5.**
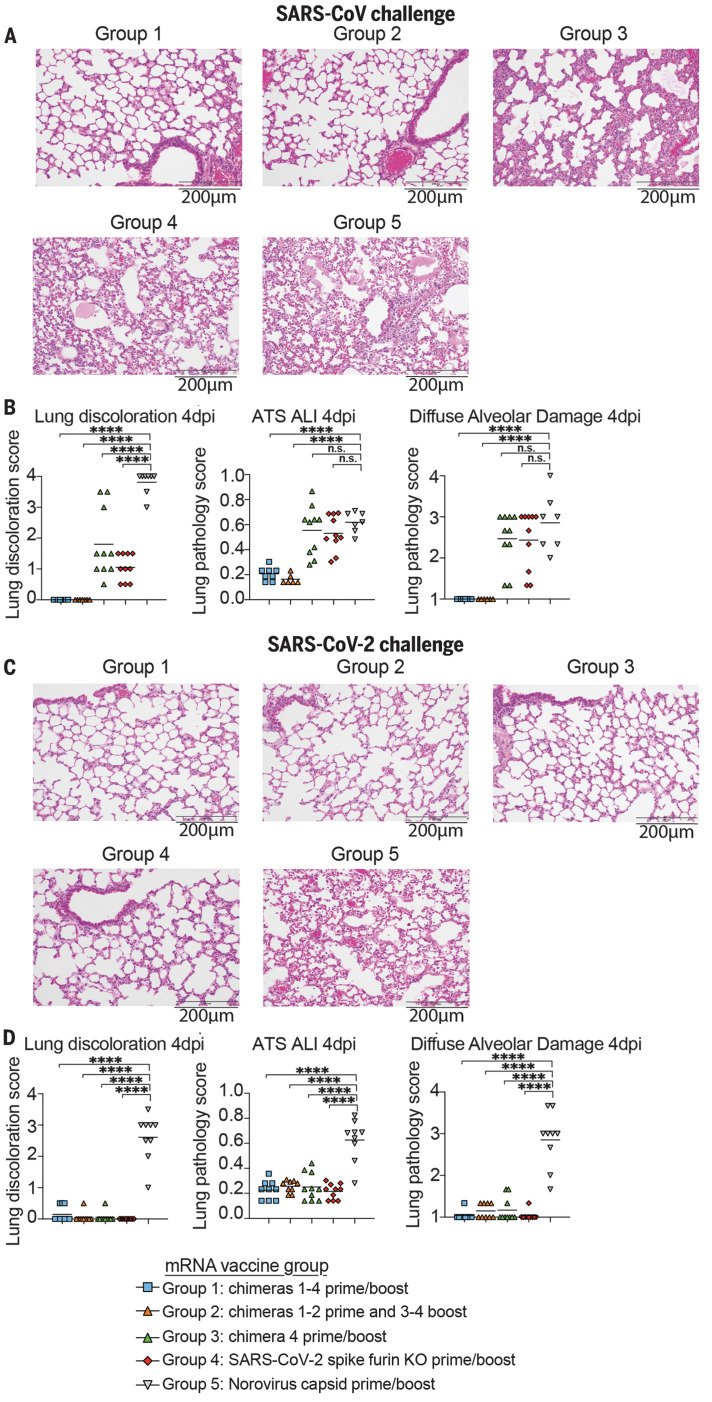
Lung pathology in vaccinated mice after SARS-CoV and SARS-CoV-2 challenge. (**A**) Hematoxylin and eosin 4 days after infection lung analysis of SARS-CoV MA15–challenged mice from the different groups: group 1, chimeras 1 to 4 prime and boost; group 2, chimeras 1 and 2 prime and 3 and 4; group 3, chimera 4 prime and boost, SARS-CoV-2 furin KO prime and boost, and norovirus capsid prime and boost. (**B**) Lung pathology quantitation in SARS-CoV MA15–challenged mice from the different groups. Macroscopic lung discoloration score, microscopic ALI score, and DAD in day 4 after infection lung tissues are shown. (**C**) Hematoxylin and eosin 4 days after infection lung analysis of SARS-CoV-2 MA10–challenged mice from the different groups. (**D**) Lung pathology measurements in SARS-CoV-2 MA10–challenged mice from the different groups. Macroscopic lung discoloration score, microscopic ALI score, and DAD in day 4 after infection lung tissues are shown. Statistical significance is reported from a one-way ANOVA after Dunnet’s multiple comparison correction. *P < 0.05, **P < 0.01, ***P < 0.001, and ****P < 0.0001.

To quantify the pathological features of acute lung injury (ALI) in mice, we used a tool from the American Thoracic Society (ATS). We similarly scored lung tissue sections for diffuse alveolar damage (DAD), the pathological hallmark of ALI (*29*, *30*). We observed significant lung pathology with both the ATS and DAD scoring tools in groups 4 and 5 vaccinated animals. By contrast, multiplexed chimeric spike vaccine formulations in groups 1 and 2 provided complete protection from lung pathology after SARS-CoV MA15 challenge (Fig. 5, A and B). Mice immunized with the SARS-CoV-2 mRNA vaccine that showed breakthrough infection with SARS-CoV MA15 developed similar lung inflammation as that of control vaccinated animals, potentially suggesting that future outbreaks of SARS-CoV may cause disease even in individuals vaccinated with SARS-CoV-2. Because eosinophilic infiltrates have been observed in vaccinated, 2003 SARS-CoV–challenged mice previously (*31*), with immunohistochemistry we analyzed lung tissues in protected versus infected animals with SARS-CoV MA15 for eosinophilic infiltrates (fig. S6). Groups 1 and 2 contained rare, scattered eosinophils in the interstitium. Group 3 showed bronchus-associated lymphoid tissue. By contrast, group 4 and group 5 contained frequent perivascular cuffs with prevalent eosinophils. All groups challenged with SARS-CoV-2 MA10 were protected against lung pathology compared with the norovirus capsid-immunized control group, supporting the hypothesis that the SARS-CoV-2 NTD present in the chimeric spike from group 3 is sufficient for protection (Fig. 5, C and D).

We measured lung proinflammatory cytokines and chemokines in the different vaccination groups. Groups 1 and 2 had baseline levels of macrophage-activating cytokines and chemokines, including interleukin-6 (IL-6), chemokine (C-C motif) ligand 2 (CCL2), IL-1α, granulocyte colony-stimulating factor (G-CSF), and CCL4, compared with group 5 after SARS-CoV MA15 challenge (fig. S7A). Group 3 and group 4 showed high and indistinguishable levels of IL-6, CCL2, IL-1α, G-CSF, and CCL4 compared with those of group 5 mice after SARS-CoV MA15 challenge. After SARS-CoV-2 MA10 challenge, group 4 and group 1 showed the lowest levels of IL-6 and G-CSF relative to that in group 5 controls (fig. S7B), and we only observed significant reductions in CCL2, IL-1α, and CCL4 lung levels in groups 3 and 4 compared with the group 5 control, despite full protection from both weight loss and lower-airway viral replication.

## Discussion

The Moderna and Pfizer/BioNTech SARS-CoV-2 mRNA-LNP vaccines were safe and efficacious against SARS-CoV-2 infections in large phase 3 efficacy human clinical trials (*32*–*34*), but there is a growing concern regarding VOCs such as South African B.1.351, which is five- to sixfold more resistant to vaccine-elicited polyclonal neutralizing antibodies (*35*). We sought to replicate the mRNA platform to formulate chimeric vaccines that specifically target distant Sarbecovirus strains. A caveat of including multiple chimeric spikes in a single shot is the potential formation of heterotrimers not present in the intended vaccine formulation. Chimera 4, which contains the RsSHC014 RBD and SARS-CoV-2 NTD and S2, elicited binding and neutralizing antibodies, and mice were fully protected from Bt-CoV RsSHC014 and SARS-CoV-2 challenge, whereas SARS-CoV-2 full length did not fully protect against RsSHC014, suggesting that CoV spike vaccines can be designed to maximize their display of protective epitopes and indicates that NTD/RBD/S2 chimeric spikes may enhance protection relative to monovalent spikes. Because the NTD, RBD, and S2 contain epitopes that are targeted by protective antibodies (*17*, *19*, *36*), modular chimeric spikes may provide a way to design CoV spikes to elicit protective immunity against three Sarbecoviruses as compared with a single Sarbecovirus by a monovalent spike. The lack of protection against WIV-1 and SARS-CoV and only partial protection against RsSHC014 challenge in SARS-CoV-2 immunized mice indicates the need for the development of universal vaccination strategies that can achieve broader coverage against preemergent bat SARS-CoV–like and SARS-CoV-2–like viruses. Despite the lower-magnitude antibody responses against SARS-CoV-2 in the chimeric spike groups, a clear advantage of our chimeric spike vaccines is the clear breadth of protection against multiclade Sarbecoviruses and SARS-CoV-2 variants compared with that from the monovalent SARS-CoV-2 vaccine. Although other strategies exist, including multiplexing mosaic Sarbecovirus RBDs (*37*) and RBDs on nanoparticles (*38*), chimeric spike mRNA-LNP vaccination can achieve broad protection by using existing manufacturing technologies and are portable to other high-risk emerging coronaviruses such as group 2C MERS-CoV–related strains. Thus, chimeric spikes can clearly protect against more than one Sarbecovirus, but it is possible that multiplexed full-length spikes may protect against Sarbecoviruses.

As previously reported with RNA recombinant viruses, live Sarbecoviruses lacking ORF7/ORF8 but containing distinct SARS-CoV-2 antigenic domains were viable, reaffirming the known interchangeability and functional plasticity of the CoV spike (*21*, *39*, *40*). Our demonstration of cross-protection against multiple Sarbecovirus strains in mice lends support to the hypothesis that universal vaccines against group 2B CoVs are likely achievable. Moving forward, it will be important to determine whether other combinations of chimeric mRNA-LNP vaccines from other SARS-like viruses are protective, elicit broad T cell responses, prevent the rapid emergence of escape viruses, elicit protective responses in nonhuman primate models of Sarbecovirus pathogenesis, and can boost Sarbecovirus protective breadth in SARS-CoV-2–vaccinated or convalescent individuals.

## Supplementary Materials

### This PDF file includes:

Materials and Methods Figs. S1 to S7 Table S1 References

### Other Supplementary Material for this manuscript includes the following:

MDAR Reproducibility Checklist
