# Supplementary material for: Chimeric spike mRNA vaccines protect against Sarbecovirus challenge in mice
Source: Science. 2021 Aug 27;373(6558):991–8. doi: 10.1126/science.abi4506 (PMC8899822; doi:10.1126/science.abi4506)
Supplement: Supplementary file 1 — Materials and Methods Figs. S1 to S7 Table S1 References [file science.abi4506_sm.pdf]

Science AAAS

science.sciencemag.org/cgi/content/full/science.abi4506/DC1

# Supplementary Material for

## Chimeric spike mRNA vaccines protect against Sarbecovirus challenge in mice

David R. Martinez*, Alexandra Schäfer, Sarah R. Leist, Gabriela De la Cruz, Ande West, Elena N. Atochina-Vasserman, Lisa C. Lindesmith, Norbert Pardi, Robert Parks, Maggie Barr, Dapeng Li, Boyd Yount, Kevin O. Saunders, Drew Weissman, Barton F. Haynes, Stephanie A. Montgomery, Ralph S. Baric*

*Corresponding author. Email: david.rafael.martinez@gmail.com (D.R.M.); rbaric@email.unc.edu (R.S.B.)

Published 22 June 2021 as *Science* First Release
DOI: 10.1126/science.abi4506

**This PDF file includes:**

Materials and Methods
Figs. S1 to S7
Table S1
Reference

**Other Supplementary Material for this manuscript includes the following:**
(available at science.sciencemag.org/content/science.abi4506/DC1)

MDAR Reproducibility Checklist

**Supplementary materials.**

## Materials and Methods

### Chimeric spike vaccine design and formulation

Chimeric spike vaccines were designed with RBD and NTD swaps to increase coverage of epidemic (SARS-CoV), pandemic (SARS-CoV-2), and high-risk pre-emergent bat CoVs (bat SARS-like HKU3-1, and bat SARS-like RsSHC014). Chimeric and monovalent spike mRNA-LNP vaccines were designed based on SARS-CoV-2 spike (S) protein sequence (Wuhan-Hu-1, GenBank: MN908947.3), SARS-CoV (urbani GenBank: AY278741), bat SARS-like CoV HKU3-1 (GenBank: DQ022305), and Bat SARS-like RsSHC014 (GenBank: KC881005). Coding sequences of full-length SARS-CoV-2 furin knockout (RRAR furin cleavage site abolished between amino acids 682-685), the four chimeric spikes, and the norovirus capsid negative control were codon-optimized, synthesized and cloned into the mRNA production plasmid mRNAs were encapsulated with LNP (*41*). Briefly, mRNAs were transcribed to contain 101 nucleotide-long poly(A) tails. mRNAs were modified with m1Ψ-5′-triphosphate (TriLink #N-1081) instead of UTP and the *in vitro* transcribed mRNAs capped using the trinucleotide cap1 analog, CleanCap (TriLink #N-7413). mRNA was purified by cellulose (Sigma-Aldrich # 11363-250G) purification. All mRNAs were analyzed by agarose gel electrophoresis and were stored at -20°C. Cellulose-purified m1Ψ-containing RNAs were encapsulated in proprietary LNPs containing adjuvant (Acuitas) using a self-assembly process as previously described wherein an ethanolic lipid mixture of ionizable cationic lipid, phosphatidylcholine, cholesterol and polyethylene glycol-lipid was rapidly mixed with an aqueous solution containing mRNA at

acidic pH. The RNA-loaded particles were characterized and subsequently stored at -80°C at a concentration of 1 mg/ml. The mean hydrodynamic diameter of these mRNA-LNP was ~80 nm with a polydispersity index of 0.02-0.06 and an encapsulation efficiency of ~95%.

**Animals, immunizations, and challenge viruses**

Eleven-month-old female BALB/c mice were purchased from Envigo (#047) and were used for all experiments. The study was carried out in accordance with the recommendations for care and use of animals by the Office of Laboratory Animal Welfare (OLAW), National Institutes of Health and the Institutional Animal Care and Use Committee (IACUC) of University of North Carolina (UNC permit no. A-3410-01). mRNA-LNP vaccines were kept frozen until right before the vaccination. Mice were immunized with a total 1μg in the prime and boost. Briefly, chimeric vaccines were mixed at 1:1 ratio for a total of 1μg when more than one chimeric spike was used or 1μg of a single spike diluted in sterile 1XPBS in a 50μl volume and were given 25μl intramuscularly in each hind leg. Equal amounts of vaccines were used to more compare the vaccines groups head-to-head. Prime and boost immunizations were given three weeks apart. Three weeks post boost, mice were bled, sera was collected for analysis, and mice were moved into the BSL3 facility for challenge experiments. Animals were housed in groups of five and fed standard chow diets. Virus inoculations were performed under anesthesia and all efforts were made to minimize animal suffering. All mice were anesthetized and infected intranasally with $1 \times 10^4$ PFU/ml of SARS-CoV MA15, $1 \times 10^4$ PFU/ml of SARS-CoV-2 MA10, $1 \times 10^4$ PFU/ml RsSHC014, $1 \times 10^4$ PFU/ml RsSHC014-MA15, $1 \times 10^5$ PFU/ml WIV-1, and $1 \times 10^4$ PFU/ml SARS-CoV-2 B.1.351-MA10 which have been described previously (*42, 43*). Mice were weighted daily and monitored for signs of clinical disease. Each challenge virus challenge

experiment encompassed 50 mice with 10 mice per vaccine group to obtain statistical power. Mouse vaccinations and challenge experiments were independently repeated twice to ensure reproducibility.

**Measurement of mouse CoV spike binding antibodies by ELISA**

Mouse serum samples from pre-immunization (pre-prime), 2 weeks post prime (pre-boost), and 3 weeks post boost were tested. A binding ELISA panel that included SARS-CoV spike Protein DeltaTM, SARS-CoV-2 (2019-nCoV) spike Protein (S1+S2 ECD, His tag), MERS-CoV, Coronavirus spike S1+S2 (Baculovirus-Insect Cells, His), HKU1 (isolate N5) spike Protein (S1+S2 ECD, His Tag), OC43 spike Protein (S1+S2 ECD, His Tag), 229E spike Protein (S1+S2 ECD, His tag) Human coronavirus (HCoV-NL63) spike Protein (S1+S2 ECD, His Tag), Pangolin CoV_GXP4L_spikeEcto2P_3C8HtS2/293F, bat CoV RsSHC014_spikeEcto2P_3C8HtS2/293F, RaTG13_spikeEcto2P_3C8HtS2/293F, and bat CoV HKU3-1 spike were tested. Indirect binding ELISAs were conducted in 384 well ELISA plates (Costar #3700) coated with 2μg/ml antigen in 0.1M sodium bicarbonate overnight at 4°C, washed and blocked with assay diluent (1XPBS containing 4% (w/v) whey protein/ 15% Normal Goat Serum/ 0.5% Tween-20/ 0.05% Sodium Azide). Serum samples were incubated for 60 minutes in three-fold serial dilutions beginning at 1:30 followed by washing with PBS/0.1% Tween-20. HRP conjugated goat anti-mouse IgG secondary antibody (SouthernBiotech 1030-05) was diluted to 1:10,000 in assay diluent without azide, incubated at for 1 hour at room temperature, washed and detected with 20µl SureBlue Reserve (KPL 53-00-03) for 15 minutes. Reactions were stopped via the addition of 20µl HCL stop solution. Plates were read at 450nm. Area under the curve (AUC) measurements were determined from binding of serial dilutions.

**ACE2 blocking ELISAs.**

Plates were coated with 2μg/ml recombinant ACE2 protein, then washed and blocked with 3% BSA in PBS. While assay plates blocked, and sera was diluted 1:25 in 1%BSA/0.05% Tween-20. Then SARS-CoV-2 spike protein was mixed with equal volumes of each sample at a final spike concentration equal to the $EC_{50}$ at which it binds to ACE2. The mixture was allowed to incubate at room temperature for 1 hour. Blocked assay plates were washed, and the serum-spike mixture was added to the assay plates for a period of 1 hour at room temperature. Plates were washed and Strep-Tactin HRP, (IBA GmbH, Cat# 2-1502-001) was added at a dilution of 1:5000 followed by TMB substrate. The extent to which antibodies were able to block the binding of spike protein to ACE2 was determined by comparing the OD of antibody samples at 450nm to the OD of samples containing spike protein only with no antibody. The following formula was used to calculate percent blocking (100-(OD sample/OD of spike only) *100).

**Measurement of neutralizing antibodies against live viruses**

Full-length SARS-CoV-2 Seattle, SARS-CoV-2 D614G, SARS-CoV-2 B.1.351, SARS-CoV-2 B.1.1.7, SARS-CoV-2 mink cluster 5, SARS-CoV, WIV-1, and RsSHC014 viruses were designed to express nanoluciferase (nLuc) and were recovered via reverse genetics. Virus titers were measured in Vero E6 USAMRIID cells, as defined by plaque forming units (PFU) per ml, in a 6-well plate format in quadruplicate biological replicates for accuracy. For the 96-well neutralization assay, Vero E6 USAMRID cells were plated at 20,000 cells per well the day prior in clear bottom black walled plates. Cells were inspected to ensure confluency on the day of assay. Serum samples were tested at a starting dilution of 1:20 and were serially diluted 3-fold up

to nine dilution spots. Serially diluted serum samples were mixed in equal volume with diluted virus. Antibody-virus and virus only mixtures were then incubated at 37°C with 5% $CO_2$ for one hour. Following incubation, serially diluted sera and virus only controls were added in duplicate to the cells at 75 PFU at 37°C with 5% $CO_2$. After 24 hours, cells were lysed, and luciferase activity was measured via Nano-Glo Luciferase Assay System (Promega) according to the manufacturer specifications. Luminescence was measured by a Spectramax M3 plate reader (Molecular Devices, San Jose, CA). Virus neutralization titers were defined as the sample dilution at which a 50% reduction in RLU was observed relative to the average of the virus control wells.

**Eosinophilic lung infiltrates staining**

To detect eosinophils, chromogenic immunohistochemistry (IHC) was performed on paraffin-embedded lung tissues that were sectioned at 4 microns. Lung tissues from vaccine groups 1-5 were analyzed for lung eosinophilic infiltration. N=8-10 lung tissues per group were analyzed. This IHC was carried out using the Leica Bond III Autostainer system. Slides were dewaxed in Bond Dewax solution (AR9222) and hydrated in Bond Wash solution (AR9590). Heat induced antigen retrieval was performed for 20 min at 100ºC in Bond-Epitope Retrieval solution 2, pH-9.0 (AR9640). After pretreatment, slides were incubated with an Eosinophil Peroxidase antibody (PA5-62200, Invitrogen) at 1:1,000 for 1h followed with Novolink Polymer (RE7260-K) secondary.  Antibody detection with 3,3'-diaminobenzidine (DAB) was performed using the Bond Intense R detection system (DS9263). Stained slides were dehydrated and coverslipped with Cytoseal 60 (8310-4, Thermo Fisher Scientific). Two positive controls (one with high and

another with low eosinophil reactivity) and a negative control (no primary antibody) were included in all staining runs.

**Lung pathology scoring**

Lung discoloration is the gross manifestation of various processes of acute lung damage, including congestion, edema, hyperemia, inflammation, and protein exudation. We used a macroscopic scoring scheme to visually score mouse lungs at the time of harvest. Acute lung injury was quantified via two separate lung pathology scoring scales: Matute-Bello and Diffuse Alveolar Damage (DAD) scoring systems. Analyses and scoring were performed by a board certified veterinary pathologist who was blinded to the treatment groups. Lung pathology slides were read and scored at 600X total magnification.

The lung injury scoring system used is from the American Thoracic Society (Matute-Bello) in order to help quantitate histological features of ALI observed in mouse models to relate this injury to human settings. In a blinded manner, three random fields of lung tissue were chosen and scored for the following: (A) neutrophils in the alveolar space (none = 0, 1–5 cells = 1, > 5 cells = 2), (B) neutrophils in the interstitial septa (none = 0, 1–5 cells = 1, > 5 cells = 2), (C) hyaline membranes (none = 0, one membrane = 1, > 1 membrane = 2), (D) Proteinaceous debris in air spaces (none = 0, one instance = 1, > 1 instance = 2), (E) alveolar septal thickening (< 2x mock thickness = 0, 2–4x mock thickness = 1, > 4x mock thickness = 2). To obtain a lung injury score per field, A–E scores were put into the following formula score = [(20x A) + (14 x B) + (7 x C) + (7 x D) + (2 x E)]/100. This formula contains multipliers that assign varying levels of importance for each phenotype of the disease state. The scores for the three fields per mouse were averaged to obtain a final score ranging from 0 to and including 1. This lung

histology scoring scale measures diffuse alveolar damage (DAD) (cellular sloughing, necrosis, hyaline membranes, etc.). Similar to the implementation of the ATS histology scoring scale, three random fields of lung tissue were scored for the following in a blinded manner: 1= absence of cellular sloughing and necrosis, 2=Uncommon solitary cell sloughing and necrosis (1–2 foci/field), 3=multifocal (3+foci) cellular sloughing and necrosis with uncommon septal wall hyalinization, or 4=multifocal ( >75% of field) cellular sloughing and necrosis with common and/or prominent hyaline membranes. The scores for the three fields per mouse were averaged to get a final DAD score per mouse. The microscope images were generated using an Olympus Bx43 light microscope and CellSense Entry v3.1 software.

**Measurement of lung cytokines**

Lung tissue was homogenized, spun down at 13,000g, and supernantant was used to measure lung cytokines using Mouse Cytokine 23-plex Assay (BioRad). Briefly, 50µl of lung homogenate supernatant was added to each well and the protocol was followed according to the manufacturer specifications. Plates were read using a MAGPIX multiplex reader (Luminex Corporation).

**Biocontainment and biosafety**

Studies were approved by the UNC Institutional Biosafety Committee approved by animal and experimental protocols in the Baric laboratory. All work described here was performed with approved standard operating procedures for SARS-CoV-2 in a biosafety level 3 (BSL-3) facility conforming to requirements recommended in the Microbiological and Biomedical Laboratories, by the U.S. Department of Health and Human Service, the U.S. Public

Health Service, and the U.S. Center for Disease Control and Prevention (CDC), and the National Institutes of Health (NIH).

**Statistics**

All statistical analyses were performed using GraphPad Prism 9. Statistical tests used in each figure are denoted in the corresponding figure legend.

**Figure S1. Chimeric and wild type spike Sarbecovirus constructs.**

(**A**) Mouse vaccination strategy using mRNA-LNPs: group 1 received chimeric spike 1, 2, 3, and 4 as the prime and boost, group 2 received chimeric spike 1, 2 as the prime and chimeric spikes 3 and 4 as the boost, group 3 received chimeric spike 4 as the prime and boost, group 4 received SARS-CoV-2 furin KO prime and boost, and group 5 received a norovirus capsid prime and boost. Different vaccine groups were separately challenged with 1) SARS-CoV MA15, 2) SARS-CoV-2 MA10, 3) RsSHC014 full-length virus, 4) RsSHC014-MA15, 5) WIV-1, and 6) SARS-CoV-2 B.1.351 MA10. (**B**) Protein expression of chimeric spikes, SARS-CoV-2 furin KO, and norovirus mRNA vaccines. The extra band between 100-150 kDa corresponds to S1. GAPDH was used as the loading control. (**C**) Nanoluciferase expression of RsSHC014/SARS-CoV-2 chimeric spike live viruses.

**Figure S2. Human common-cold CoV ELISA binding responses in chimeric and monovalent SARS-CoV-2 spike mRNA-LNP-vaccinated mice.** Pre-immunization, post prime, and post boost binding to (**A**) HCoV-HKU1 spike, (**B**) HCoV-OC43 spike, (**C**) HCoV-229E spike, and (**D**) HCoV-NL63 spike. Statistical significance for the binding and blocking responses is reported from a Kruskal-Wallis test after Dunnett's multiple comparison correction. $^{*}p < 0.05$, $^{**}p < 0.01$, $^{***}p < 0.001$, and $^{****}p < 0.0001$.

**Figure S3. Comparison of neutralizing antibody activity of CoV mRNA-LNP vaccines against Sarbecoviruses.** (**A**) Group 1 neutralizing antibody responses against SARS-CoV-2, SARS-CoV, RsSHC014, and WIV-1 and (**B**) fold-change of SARS-CoV, RsSHC014, and WIV-1 neutralizing antibodies relative to SARS-CoV-2. (**C**) Group 2 neutralizing antibody responses

against SARS-CoV-2, SARS-CoV, RsSHC014, and WIV-1 and (**D**) fold-change of SARS-CoV, RsSHC014, and WIV-1 neutralizing antibodies relative to SARS-CoV-2. (**E**) Group 3 neutralizing antibody responses against SARS-CoV-2, SARS-CoV, RsSHC014, and WIV-1 and (**F**) fold-change of SARS-CoV, RsSHC014, and WIV-1 neutralizing antibodies relative to SARS-CoV-2. (**G**) Group 4 neutralizing antibody responses against SARS-CoV-2, SARS-CoV, RsSHC014, and WIV-1 and (**H**) fold-change of SARS-CoV, RsSHC014, and WIV-1 neutralizing antibodies relative to SARS-CoV-2.

**Figure S4. *In vivo* protection against Bt-CoV challenge by chimeric spikes mRNA-vaccines.** (**A**) Percent starting weight from the different vaccine groups of mice challenged with full-length RsSHC014. (**B**) RsSHC014 lung viral titers in mice from the distinct vaccine groups. (**C**) RsSHC014 nasal turbinate titers in mice from the different immunization groups. (**D**) Percent starting weight from the different vaccine groups of mice challenged with RsSHC014-MA15. (**E**) RsSHC014-MA15 lung viral titers in mice from the distinct vaccine groups. (**F**) RsSHC014-MA15 nasal turbinate titers in mice from the different immunization groups. Statistical significance is reported from a one-way ANOVA after Tukey's multiple comparison correction. $^{*}p < 0.05$, $^{**}p < 0.01$, $^{***}p < 0.001$, and $^{****}p < 0.0001$.

**Figure S5. Survival analysis of immunized mice challenged with Sarbecoviruses.** (**A**) Survival analysis at day 4 post infection from immunized mice infected with SARS-CoV MA15, (**B**) SARS-CoV-2 MA10, (**C**) Survival analysis at day 7 post infection from immunized mice infected with SARS-CoV-2 MA10, and (**D**) RsSHC014-MA15. Statistical significance is reported from a Mantel-Cox test.

**Figure S6. Detection of eosinophilic infiltrates in SARS-CoV MA15 challenged mice.** (**A**) Group 1: rare scattered individual eosinophils in the interstitium with some small perivascular cuffs that lack eosinophils. (**B**) Group 2: Bronchiolar cuffs of leukocytes with rare eosinophils. (**C**) Group 3: Hyperplastic bronchus-associated lymphoid tissue (BALT) with rare eosinophils. (**D**) Group 4: frequent perivascular cuffs that contain eosinophils. (**E**) Group 5: frequent eosinophils in perivascular cuffs.

**Figure S7. Lung cytokine analysis in Sarbecovirus-challenged mice.** CCL2, IL-1α, G-SCF, and CCL4 in (**A**) SARS-CoV-infected mice and in (**B**) SARS-CoV-2-infected mice. Statistical significance for the binding and blocking responses is reported from a Kruskal-Wallis test after Dunnett's multiple comparison correction. *$p < 0.05$, **$p < 0.01$, ***$p < 0.001$, and ****$p < 0.0001$.

**Table S1: Amino acid sequences of chimeric spikes.**

Figure S1

A

Immunization strategy and challenge viruses in the different vaccine groups.

| Vaccination group | Day 0 prime | Day 21 boost | Day 55 post prime challenge viruses |
|---|---|---|---|
| Group 1 | Chimera 1, 2, 3, 4 | Chimera 1, 2, 3, 4 | 1) SARS-CoV MA15, 2) SARS-CoV-2 MA10<br>3) RsSHC014, 4) RsSHC014-MA15<br>5) WIV-1, 6) SARS-CoV-2 B.1.351-MA10 |
| Group 2 | Chimera 1, 2 | Chimera 3, 4 | 1) SARS-CoV MA15, 2) SARS-CoV-2 MA10<br>3) RsSHC014, 4) RsSHC014-MA15<br>5) WIV-1, 6) SARS-CoV-2 B.1.351-MA10 |
| Group 3 | Chimera 4 | Chimera 4 | 1) SARS-CoV MA15, 2) SARS-CoV-2 MA10<br>3) RsSHC014, 4) RsSHC014-MA15<br>5) WIV-1, 6) SARS-CoV-2 B.1.351-MA10 |
| Group 4 | SARS-CoV-2<br>furin knockout | SARS-CoV-2<br>furin knockout | 1) SARS-CoV MA15, 2) SARS-CoV-2 MA10<br>3) RsSHC014, 4) RsSHC014-MA15<br>5) WIV-1, 6) SARS-CoV-2 B.1.351-MA10 |
| Group 5 | Norovirus capsid | Norovirus capsid | 1) SARS-CoV MA15, 2) SARS-CoV-2 MA10<br>3) RsSHC014, 4) RsSHC014-MA15<br>5) WIV-1, 6) SARS-CoV-2 B.1.351-MA10 |

B

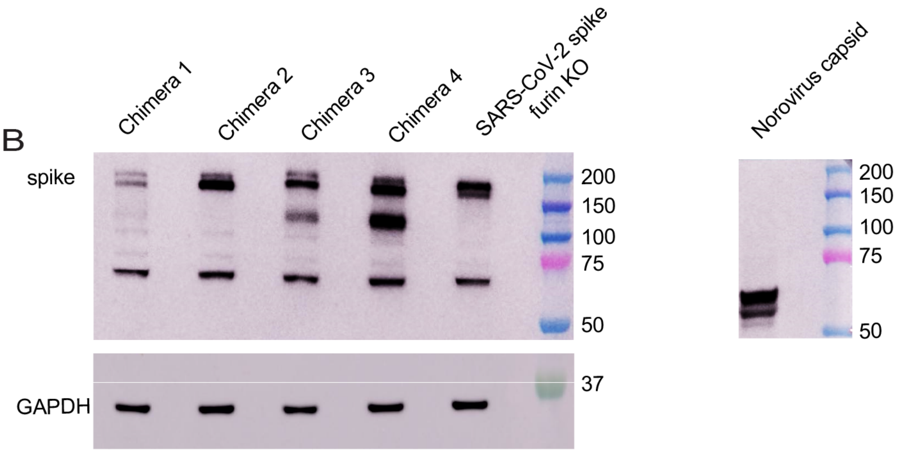


C

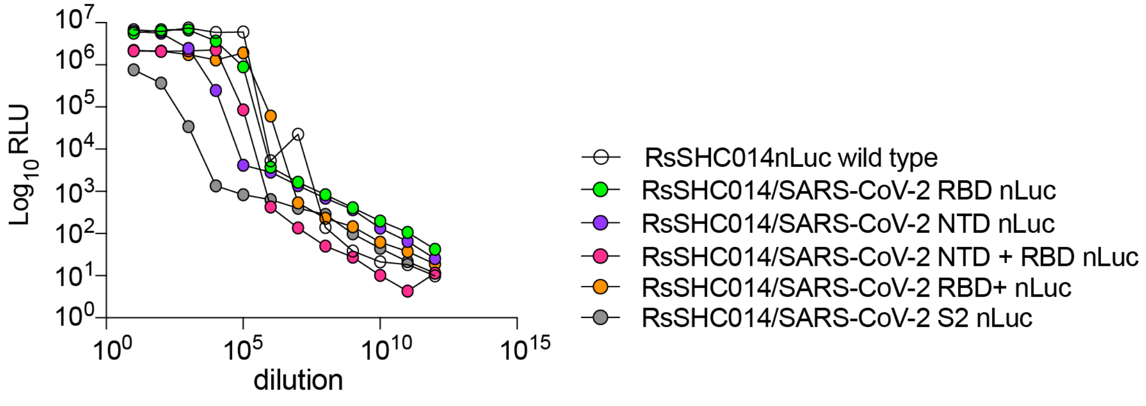

Figure S2

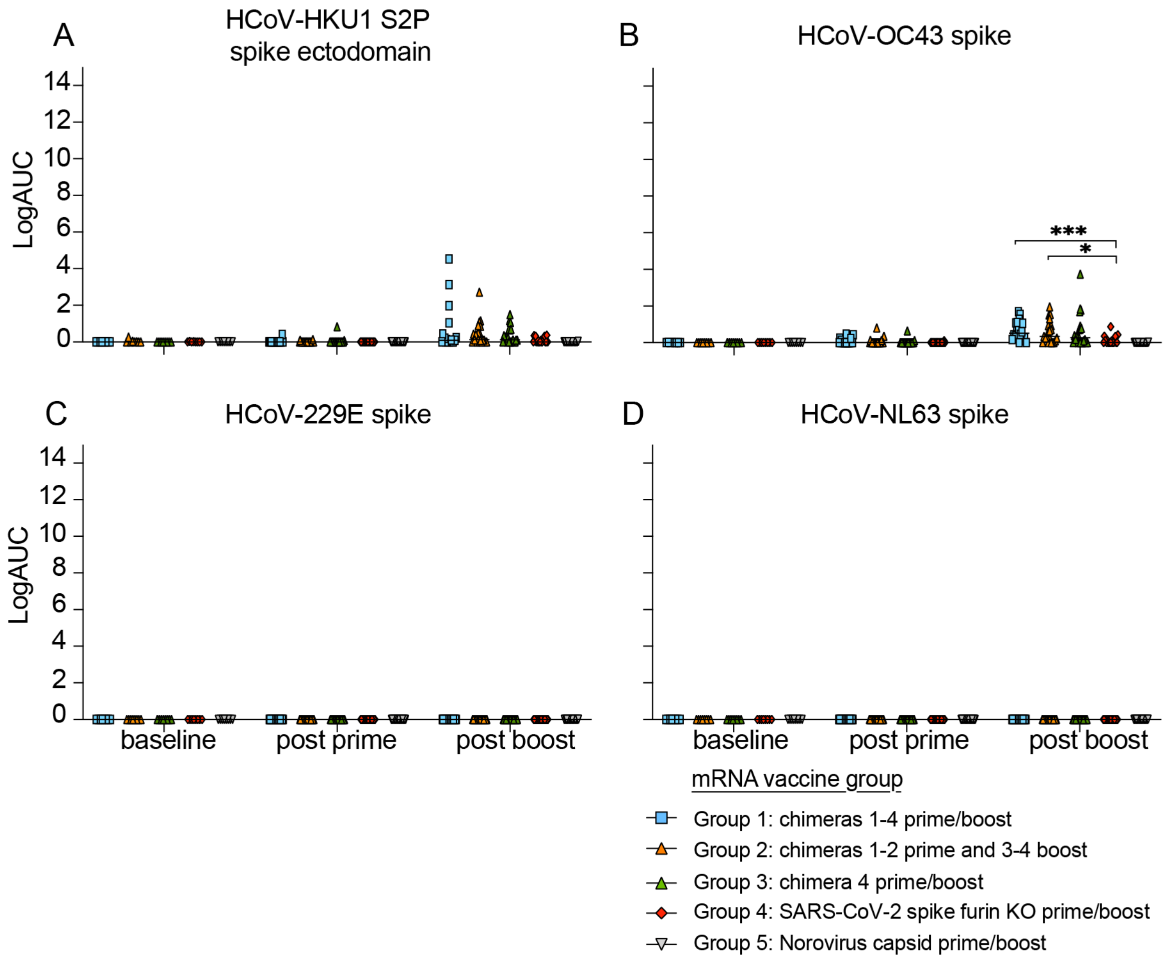
A
HCoV-HKU1 S2P spike ectodomain
B
HCoV-OC43 spike
C
HCoV-229E spike
D
HCoV-NL63 spike
LogAUC
0
2
4
6
8
10
12
14
***
*
baseline
post prime
post boost
mRNA vaccine group
Group 1: chimeras 1-4 prime/boost
Group 2: chimeras 1-2 prime and 3-4 boost
Group 3: chimera 4 prime/boost
Group 4: SARS-CoV-2 spike furin KO prime/boost
Group 5: Norovirus capsid prime/boost

Figure S3

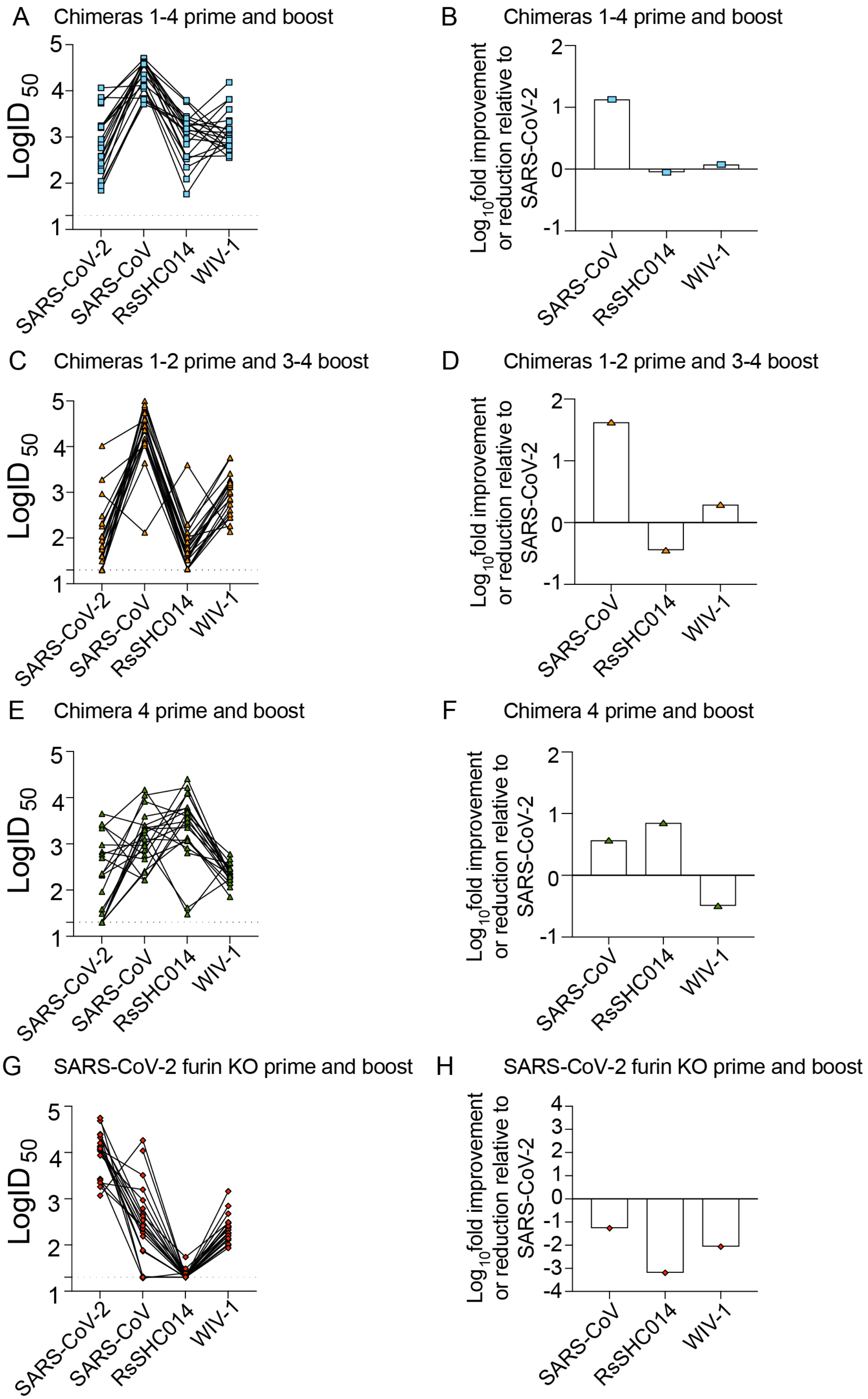
A
Chimeras 1-4 prime and boost
B
Chimeras 1-4 prime and boost
C
Chimeras 1-2 prime and 3-4 boost
D
Chimeras 1-2 prime and 3-4 boost
E
Chimera 4 prime and boost
F
Chimera 4 prime and boost
G
SARS-CoV-2 furin KO prime and boost
H
SARS-CoV-2 furin KO prime and boost
LogID 50
Log10fold improvement or reduction relative to SARS-CoV-2
SARS-CoV-2
SARS-CoV
RsSHC014
WIV-1

Figure S4

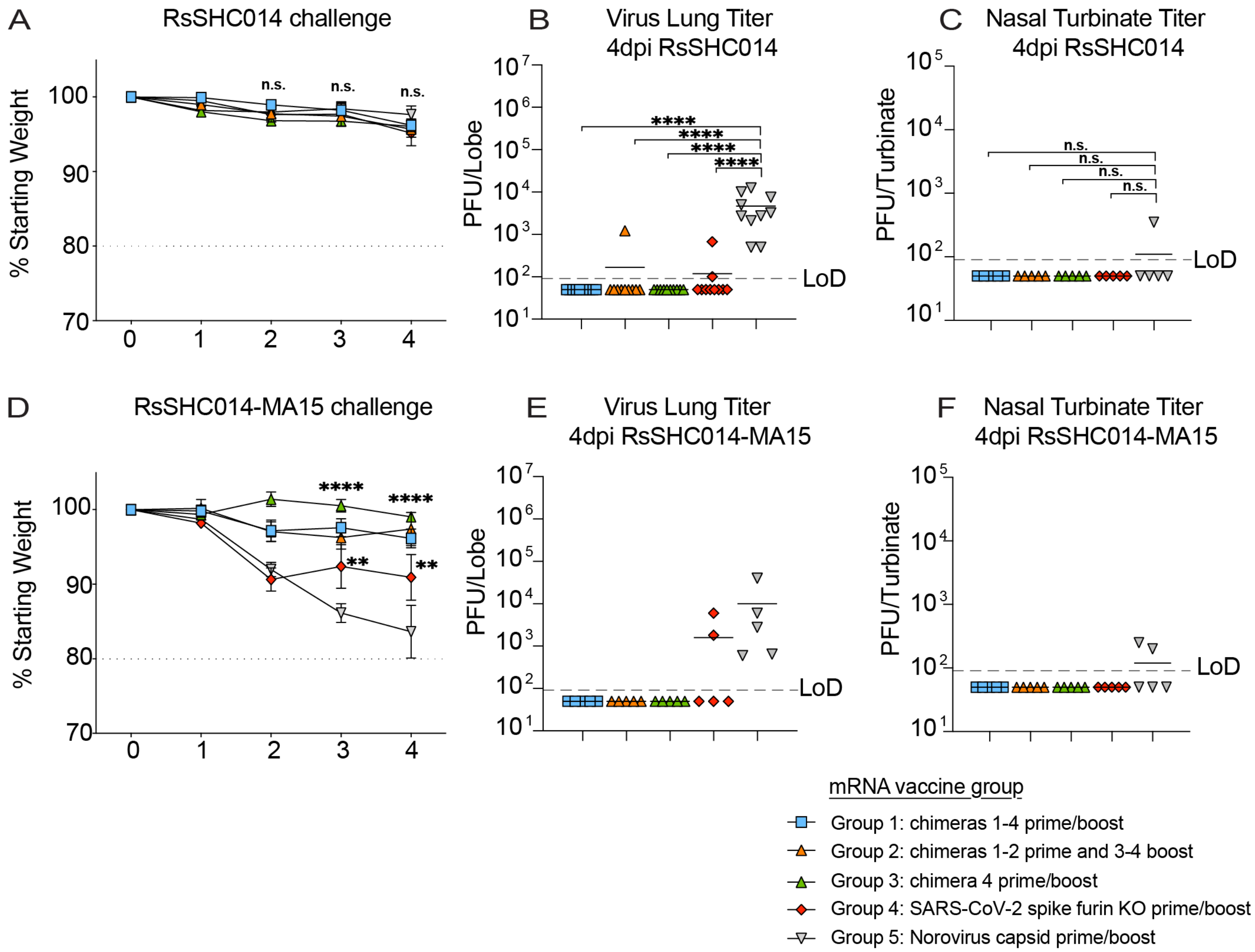
A
RsSHC014 challenge
% Starting Weight
n.s.
B
Virus Lung Titer
4dpi RsSHC014
PFU/Lobe
****
LoD
C
Nasal Turbinate Titer
4dpi RsSHC014
PFU/Turbinate
n.s.
D
RsSHC014-MA15 challenge
****
**
E
Virus Lung Titer
4dpi RsSHC014-MA15
F
Nasal Turbinate Titer
4dpi RsSHC014-MA15
mRNA vaccine group
Group 1: chimeras 1-4 prime/boost
Group 2: chimeras 1-2 prime and 3-4 boost
Group 3: chimera 4 prime/boost
Group 4: SARS-CoV-2 spike furin KO prime/boost
Group 5: Norovirus capsid prime/boost

Figure S5

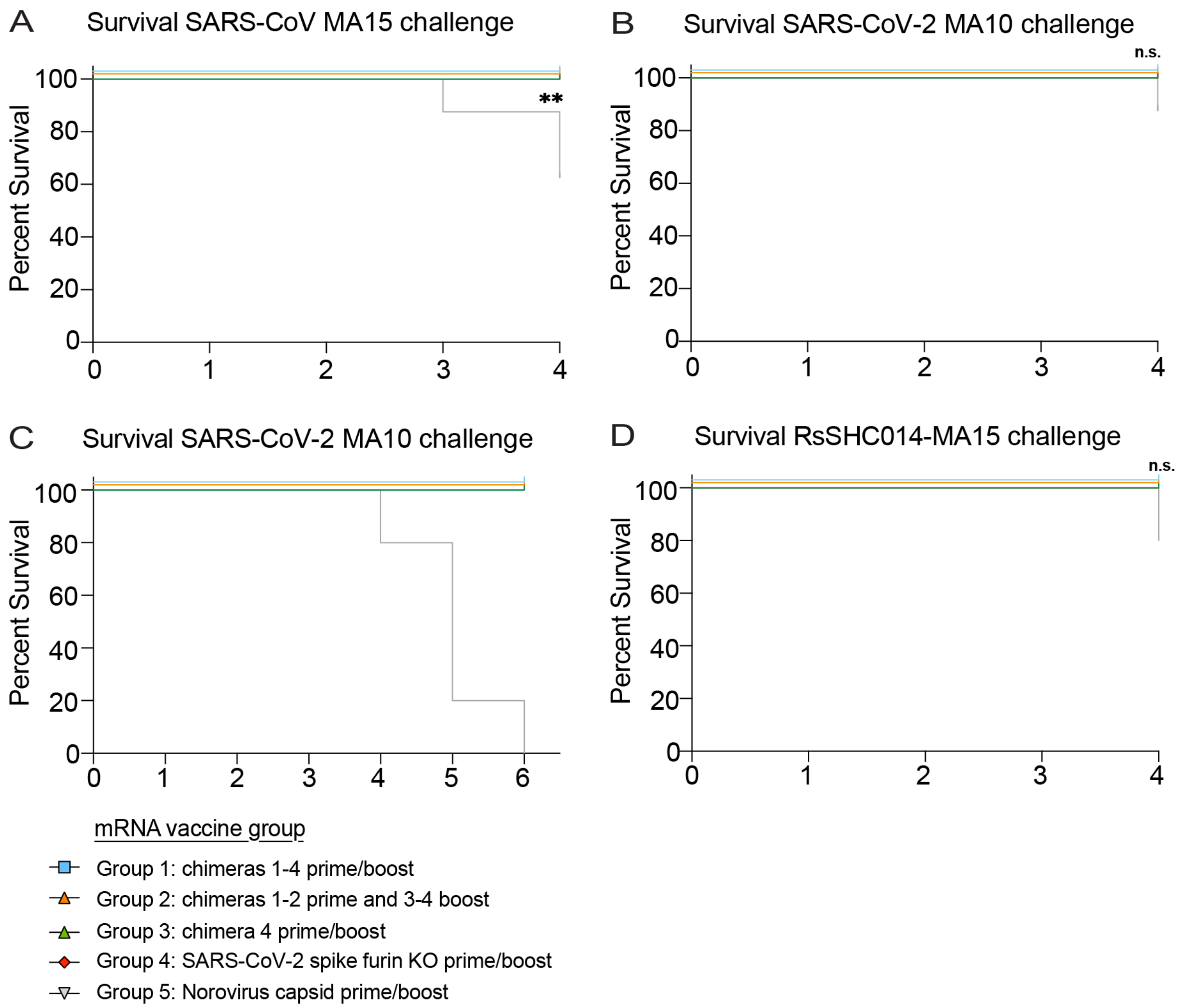
A
Survival SARS-CoV MA15 challenge
Percent Survival
**
B
Survival SARS-CoV-2 MA10 challenge
Percent Survival
n.s.
C
Survival SARS-CoV-2 MA10 challenge
Percent Survival
D
Survival RsSHC014-MA15 challenge
Percent Survival
n.s.
mRNA vaccine group
Group 1: chimeras 1-4 prime/boost
Group 2: chimeras 1-2 prime and 3-4 boost
Group 3: chimera 4 prime/boost
Group 4: SARS-CoV-2 spike furin KO prime/boost
Group 5: Norovirus capsid prime/boost

Figure S6

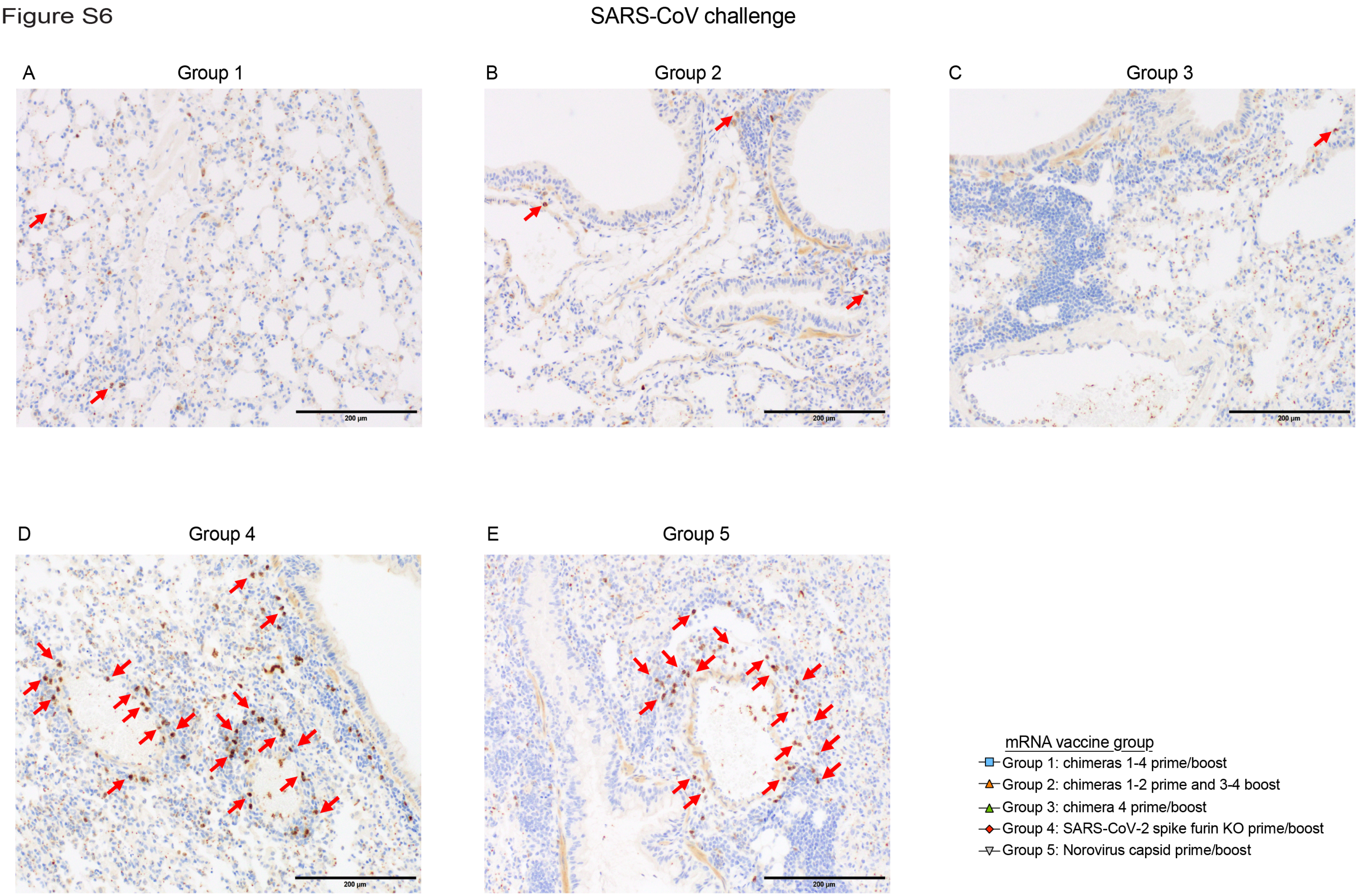
SARS-CoV challenge
A
Group 1
B
Group 2
C
Group 3
D
Group 4
E
Group 5
200 µm
mRNA vaccine group
Group 1: chimeras 1-4 prime/boost
Group 2: chimeras 1-2 prime and 3-4 boost
Group 3: chimera 4 prime/boost
Group 4: SARS-CoV-2 spike furin KO prime/boost
Group 5: Norovirus capsid prime/boost

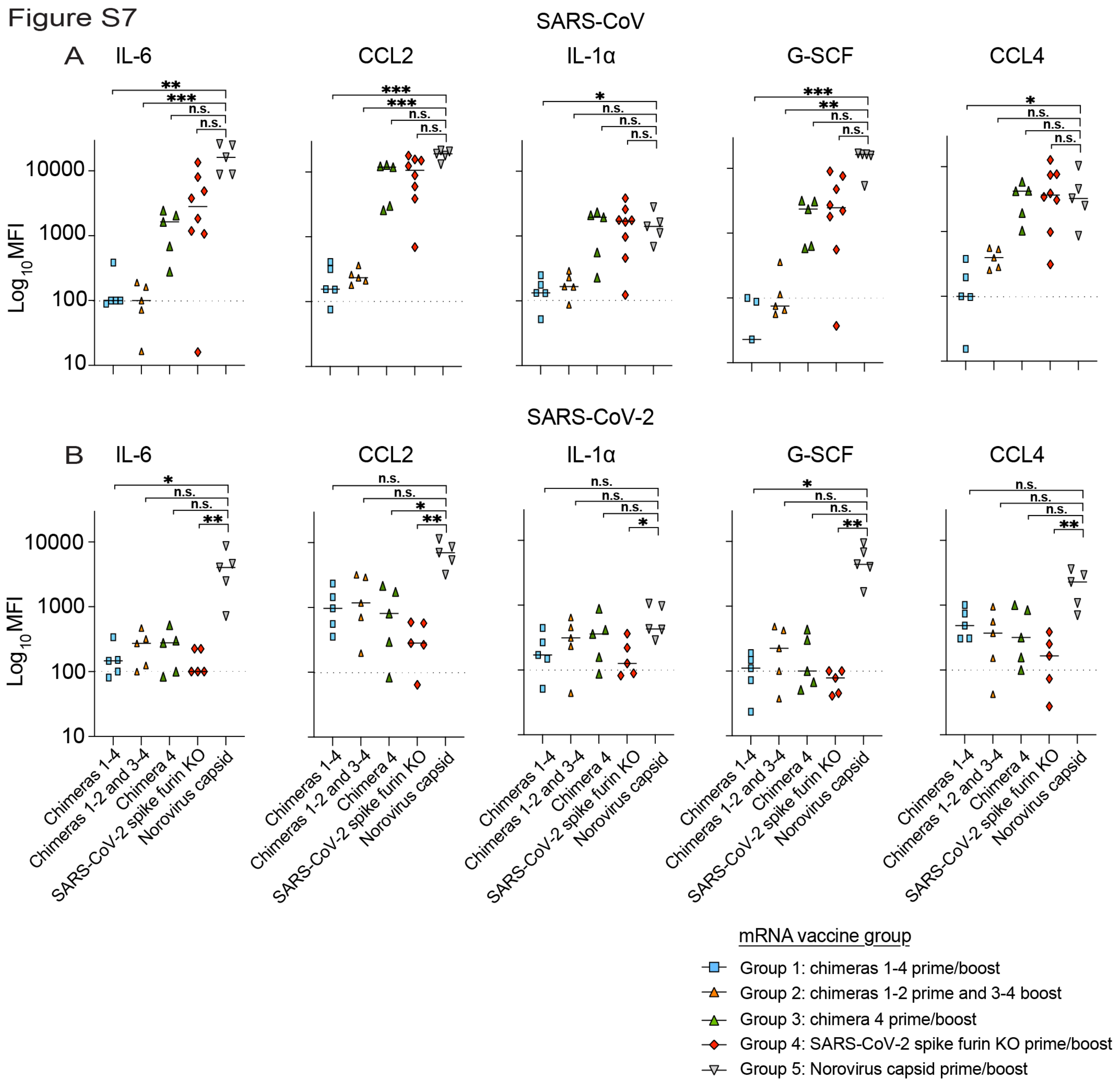

Figure S7
SARS-CoV
A
IL-6
CCL2
IL-1α
G-SCF
CCL4
**
***
*
n.s.
Log10MFI
10000
1000
100
10
SARS-CoV-2
B
Chimeras 1-4
Chimeras 1-2 and 3-4
Chimera 4
SARS-CoV-2 spike furin KO
Norovirus capsid
mRNA vaccine group
Group 1: chimeras 1-4 prime/boost
Group 2: chimeras 1-2 prime and 3-4 boost
Group 3: chimera 4 prime/boost
Group 4: SARS-CoV-2 spike furin KO prime/boost
Group 5: Norovirus capsid prime/boost

**Table S1: Amino acid sequences of chimeric spikes**

| | |
|---|---|
| Chimera 1: | MAISGVPVLGFFIIAVLMSAQESWAGIISRKPQPKMAQVSSSRRGVYYNDDIFRSDVLHLTQDYFLPFDSNLTQYFSLN<br>VDSDRYTYFDNPILDFGDGVYFAATEKSNVIRGWIFGSSFDNTTQSAVIVNNSTHIIIRVCNFNLCKEPMYTVSRGTQQ<br>NAWVYQSAFNCTYDRVEKSFQLDTTPKTGNFKDLREYVFKNRDGFLSVYQTYTAVNLPRGLPTGFSVLKPILKLPFGI<br>NITSYRVVMAMFSQTTSNFLPESAAYYVGNLKYSTFMLRFNENGTITDAVDCSQNPLAELKCTIKNFTVEKGIYQTSN<br>FRVQPTESIVRFPNITNLCPFGEVFNATKFPSVYAWERKKISNCVADYSVLYNSTFFSTFKCYGVSATKLNDLCFSNVY<br>ADSFVVKGDDVRQIAPGQTGVIADYNYKLPDDFMGCVLAWNTRNIDATSTGNYNYKYRYLRHGKLRPFERDISNVPF<br>SPDGKPCTPPALNCYWPLNDYGFYTTTGIGYQPYRVVVLSFELLNAPATVCGPKKSTNLVKNKCVNFNFNGLTGTGV<br>LTESNKKFLPFQQFGRDIADTTDAVRDPQTLEILDITPCSFGGVSVITPGTNTSNQVAVLYQDVNCTEVPVAIHADQLT<br>PTWRVYSTGSNVFQTRAGCLIGAEHVNNSYECDIPIGAGICASYQTQTNSPRRARSVASQSIIAYTMSLGAENSVAYSN<br>NSIAIPTNFTISVTTEILPVSMTKTSVDCTMYICGDSTECSNLLLQYGSFCTQLNRALTGIAVEQDKNTQEVFAQVKQIY<br>KTPPIKDFGGFNFSQILPDPSKPSKRSFIEDLLFNKVTLADAGFIKQYGDCLGDIAARDLICAQKFNGLTVLPPLLTDEMI<br>AQYTSALLAGTITSGWTFGAGAALQIPFAMQMAYRFNGIGVTQNVLYENQKLIANQFNSAIGKIQDSLSSTASALGKL<br>QDVVNQNAQALNTLVKQLSSNFGAISSVLNDILSRLDKVEAEVQIDRLITGRLQSLQTYVTQQLIRAAEIRASANLAAT<br>KMSECVLGQSKRVDFCGKGYHLMSFPQSAPHGVVFLHVTYVPAQEKNFTTAPAICHDGKAHFPREGVFVSNGTHWF<br>VTQRNFYEPQIITTDNTFVSGNCDVVIGIVNNTVYDPLQPELDSFKEELDKYFKNHTSPDVDLGDISGINASVVNIQKEI<br>DRLNEVAKNLNESLIDLQELGKYEQYIKWPWYIWLGFIAGLIAIVMVTIMLCCMTSCCSCLKGCCSCGSCCKFDEDDS<br>EPVLKGVKLHYT |
| Chimera 2: | MAISGVPVLGFFIIAVLMSAQESWASDLDRCTTFDDVQAPNYTQHTSSMRGVYYPDEIFRSDTLYLTQDLFLPFYSNV<br>TGFHTINHTFGNPVIPFKDGIYFAATEKSNVVRGWVFGSTMNNKSQSVIIINNSTNVVIRACNFELCDNPFFAVSKPMGT<br>QTHTMIFDNAFNCTFEYISDAFSLDVSEKSGNFKHLREFVFKNKDGFLYVYKGYQPIDVVRDLPSGFNTLKPIFKLPLGI<br>NITNFRAILTAFSPAQDIWGTSAAAYFVGYLKPTTFMLKYDENGTITDAVDCSQNPLAELKCSVKSFEIDKGIYQTSNF<br>RVVPSGDVVRFPNITNLCPFGEVFNATRFASVYAWNRKRISNCVADYSVLYNSASFSTFKCYGVSPTKLNDLCFTNVY<br>ADSFVIRGDEVRQIAPGQTGKIADYNYKLPDDFTGCVIAWNSNNLDSKVGGNYNYLYRLFRKSNLKPFERDISTEIYQA<br>GSTPCNGVEGFNCYFPLQSYGFQPTNGVGYQPYRVVVLSFELLHAPATVCGPKLSTDLIKNQCVNFNFNGLTGTGVLT<br>PSSKRFQPFQQFGRDVSDFTDSVRDPKTSEILDISPCSFGGVSVITPGTNASSEVAVLYQDVNCTDVSTAIHADQLTPAW<br>RIYSTGNNVFQTQAGCLIGAEHVDTSYECDIPIGAGICASYHTVSLLRSTSQKSIVAYTMSLGADSSIAYSNNTIAIPTNF<br>SISITTEVMPVSMAKTSVDCNMYICGDSTECANLLLQYGSFCTQLNRALSGIAAEQDRNTREVFAQVKQMYKTPTLKY<br>FGGFNFSQILPDPLKPTKRSFIEDLLFNKVTLADAGFMKQYGECLGDINARDLICAQKFNGLTVLPPLLTDDMIAAYTA<br>ALVSGTATAGWTFGAGAALQIPFAMQMAYRFNGIGVTQNVLYENQKQIANQFNKAISQIQESLTTTSTALGKLQDVV<br>NQNAQALNTLVKQLSSNFGAISSVLNDILSRLDKVEAEVQIDRLITGRLQSLQTYVTQQLIRAAEIRASANLAATKMSE<br>CVLGQSKRVDFCGKGYHLMSFPQAAPHGVVFLHVTYVPSQERNFTTAPAICHEGKAYFPREGVFVFNGTSWFITQRNF<br>FSPQIITTDNTFVSGNCDVVIGIINNTVYDPLQPELDSFKEELDKYFKNHTSPDVDLGDISGINASVVNIQKEIDRLNEVA<br>KNLNESLIDLQELGKYEQYIKWPWYVWLGFIAGLIAIVMVTILLCCMTSCCSCLKGACSCGSCCKFDEDDSEPVLKGV<br>KLHYT |
| Chimera 3: | MAISGVPVLGFFIIAVLMSAQESWAVNLTTRTQLPPAYTNSFTRGVYYPDKVFRSSVLHSTQDLFLPFFSNVTWFHAIH<br>VSGTNGTKRFDNPVLPFNDGVYFASTEKSNIIRGWIFGTTLDSKTQSLLIVNNATNVVIKVCEFQFCNDPFLGVYYHKN<br>NKSWMESEFRVYSSANNCTFEYVSQPFLMDLEGKQGNFKNLREFVFKNIDGYFKIYSKHTPINLVRDLPQGFSALEPL<br>VDLPIGINITRFQTLLALHRSYLTPGDSSSGWTAGAAAYYVGYLQPRTFLLKYNENGTITDAVDCALDPLSETKCTLKS<br>FTVEKGIYQTSNFRVQPTESIVRFPNITNLCPFGEVFNATKFPSVYAWERKKISNCVADYSVLYNSTFFSTFKCYGVSAT<br>KLNDLCFSNVYADSFVVKGDDVRQIAPGQTGVIADYNYKLPDDFMGCVLAWNTRNIDATSTGNYNYKYRYLRHGKL<br>RPFERDISNVPFSPDGKPCTPPALNCYWPLNDYGFYTTTGIGYQPYRVVVLSFELLNAPATVCGPKKSTNLVKNKCVN<br>FNFNGLTGTGVLTESNKKFLPFQQFGRDIADTTDAVRDPQTLEILDITPCSFGGVSVITPGTNTSNQVAVLYQDVNCTE<br>VPVAIHADQLTPTWRVYSTGSNVFQTRAGCLIGAEHVNNSYECDIPIGAGICASYQTQTNSPRRARSVASQSIIAYTMSL<br>GAENSVAYSNNSIAIPTNFTISVTTEILPVSMTKTSVDCTMYICGDSTECSNLLLQYGSFCTQLNRALTGIAVEQDKNTQ<br>EVFAQVKQIYKTPPIKDFGGFNFSQILPDPSKPSKRSFIEDLLFNKVTLADAGFIKQYGDCLGDIAARDLICAQKFNGLT<br>VLPPLLTDEMIAQYTSALLAGTITSGWTFGAGAALQIPFAMQMAYRFNGIGVTQNVLYENQKLIANQFNSAIGKIQDS<br>LSSTASALGKLQDVVNQNAQALNTLVKQLSSNFGAISSVLNDILSRLDKVEAEVQIDRLITGRLQSLQTYVTQQLIRAA<br>EIRASANLAATKMSECVLGQSKRVDFCGKGYHLMSFPQSAPHGVVFLHVTYVPAQEKNFTTAPAICHDGKAHFPREG<br>VFVSNGTHWFVTQRNFYEPQIITTDNTFVSGNCDVVIGIVNNTVYDPLQPELDSFKEELDKYFKNHTSPDVDLGDISGI<br>NASVVNIQKEIDRLNEVAKNLNESLIDLQELGKYEQYIKWPWYIWLGFIAGLIAIVMVTIMLCCMTSCCSCLKGCCSCG<br>SCCKFDEDDSEPVLKGVKLHYT |
| Chimera 4: | MAISGVPVLGFFIIAVLMSAQESWAVNLTTRTQLPPAYTNSFTRGVYYPDKVFRSSVLHSTQDLFLPFFSNVTWFHAIH<br>VSGTNGTKRFDNPVLPFNDGVYFASTEKSNIIRGWIFGTTLDSKTQSLLIVNNATNVVIKVCEFQFCNDPFLGVYYHKN<br>NKSWMESEFRVYSSANNCTFEYVSQPFLMDLEGKQGNFKNLREFVFKNIDGYFKIYSKHTPINLVRDLPQGFSALEPL<br>VDLPIGINITRFQTLLALHRSYLTPGDSSSGWTAGAAAYYVGYLQPRTFLLKYNENGTITDAVDCALDPLSETKCTLKS<br>FTVEKGIYQTSNFRVQPTESIVRFPNITNLCPFGEVFNATTFPSVYAWERKRISNCVADYSVLYNSTSFSTFKCYGVSAT<br>KLNDLCFSNVYADSFVVKGDDVRQIAPGQTGVIADYNYKLPDDFLGCVLAWNTNSKDSSTSGNYNYLYRWVRRSKL<br>NPYERDLSNDIYSPGGQSCSAVGPNCYNPLRPYGFFTTAGVGHQPYRVVVLSFELLNAPATVCGPKKSTNLVKNKCVN<br>FNFNGLTGTGVLTESNKKFLPFQQFGRDIADTTDAVRDPQTLEILDITPCSFGGVSVITPGTNTSNQVAVLYQDVNCTE<br>VPVAIHADQLTPTWRVYSTGSNVFQTRAGCLIGAEHVNNSYECDIPIGAGICASYQTQTNSPRRARSVASQSIIAYTMSL<br>GAENSVAYSNNSIAIPTNFTISVTTEILPVSMTKTSVDCTMYICGDSTECSNLLLQYGSFCTQLNRALTGIAVEQDKNTQ<br>EVFAQVKQIYKTPPIKDFGGFNFSQILPDPSKPSKRSFIEDLLFNKVTLADAGFIKQYGDCLGDIAARDLICAQKFNGLT<br>VLPPLLTDEMIAQYTSALLAGTITSGWTFGAGAALQIPFAMQMAYRFNGIGVTQNVLYENQKLIANQFNSAIGKIQDS<br>LSSTASALGKLQDVVNQNAQALNTLVKQLSSNFGAISSVLNDILSRLDKVEAEVQIDRLITGRLQSLQTYVTQQLIRAA<br>EIRASANLAATKMSECVLGQSKRVDFCGKGYHLMSFPQSAPHGVVFLHVTYVPAQEKNFTTAPAICHDGKAHFPREG<br>VFVSNGTHWFVTQRNFYEPQIITTDNTFVSGNCDVVIGIVNNTVYDPLQPELDSFKEELDKYFKNHTSPDVDLGDISGI<br>NASVVNIQKEIDRLNEVAKNLNESLIDLQELGKYEQYIKWPWYIWLGFIAGLIAIVMVTIMLCCMTSCCSCLKGCCSCG<br>SCCKFDEDDSEPVLKGVKLHYT |

## References and Notes


1. J. D. Cherry, P. Krogstad, SARS: The first pandemic of the 21st century. *Pediatr. Res.* **56**, 1–5 (2004). doi:10.1203/01.PDR.0000129184.87042.FC Medline

2. A. M. Zaki, S. van Boheemen, T. M. Bestebroer, A. D. Osterhaus, R. A. Fouchier, Isolation of a novel coronavirus from a man with pneumonia in Saudi Arabia. *N. Engl. J. Med.* **367**, 1814–1820 (2012). doi:10.1056/NEJMoa1211721 Medline

3. C. I. Paules, H. D. Marston, A. S. Fauci, Coronavirus Infections-More Than Just the Common Cold. *JAMA* **323**, 707–708 (2020). doi:10.1001/jama.2020.0757 Medline

4. P. Zhou, X.-L. Yang, X.-G. Wang, B. Hu, L. Zhang, W. Zhang, H.-R. Si, Y. Zhu, B. Li, C.-L. Huang, H.-D. Chen, J. Chen, Y. Luo, H. Guo, R.-D. Jiang, M.-Q. Liu, Y. Chen, X.-R. Shen, X. Wang, X.-S. Zheng, K. Zhao, Q.-J. Chen, F. Deng, L.-L. Liu, B. Yan, F.-X. Zhan, Y.-Y. Wang, G.-F. Xiao, Z.-L. Shi, A pneumonia outbreak associated with a new coronavirus of probable bat origin. *Nature* **579**, 270–273 (2020). doi:10.1038/s41586-020-2012-7 Medline

5. Coronaviridae Study Group of the International Committee on Taxonomy of Viruses, The species Severe acute respiratory syndrome-related coronavirus: Classifying 2019-nCoV and naming it SARS-CoV-2. *Nat. Microbiol.* **5**, 536–544 (2020). doi:10.1038/s41564-020-0695-z Medline

6. V. D. Menachery, B. L. Yount Jr., K. Debbink, S. Agnihothram, L. E. Gralinski, J. A. Plante, R. L. Graham, T. Scobey, X.-Y. Ge, E. F. Donaldson, S. H. Randell, A. Lanzavecchia, W. A. Marasco, Z.-L. Shi, R. S. Baric, A SARS-like cluster of circulating bat coronaviruses shows potential for human emergence. *Nat. Med.* **21**, 1508–1513 (2015). doi:10.1038/nm.3985 Medline

7. V. D. Menachery, B. L. Yount Jr., A. C. Sims, K. Debbink, S. S. Agnihothram, L. E. Gralinski, R. L. Graham, T. Scobey, J. A. Plante, S. R. Royal, J. Swanstrom, T. P. Sheahan, R. J. Pickles, D. Corti, S. H. Randell, A. Lanzavecchia, W. A. Marasco, R. S. Baric, SARS-like WIV1-CoV poised for human emergence. *Proc. Natl. Acad. Sci. U.S.A.* **113**, 3048–3053 (2016). doi:10.1073/pnas.1517719113 Medline

8. B. Hu, L.-P. Zeng, X.-L. Yang, X.-Y. Ge, W. Zhang, B. Li, J.-Z. Xie, X.-R. Shen, Y.-Z. Zhang, N. Wang, D.-S. Luo, X.-S. Zheng, M.-N. Wang, P. Daszak, L.-F. Wang, J. Cui, Z.-L. Shi, Discovery of a rich gene pool of bat SARS-related coronaviruses provides new insights into the origin of SARS coronavirus. *PLOS Pathog.* **13**, e1006698 (2017). doi:10.1371/journal.ppat.1006698 Medline

9. T. P. Sheahan, A. C. Sims, R. L. Graham, V. D. Menachery, L. E. Gralinski, J. B. Case, S. R. Leist, K. Pyrc, J. Y. Feng, I. Trantcheva, R. Bannister, Y. Park, D. Babusis, M. O. Clarke, R. L. Mackman, J. E. Spahn, C. A. Palmiotti, D. Siegel, A. S. Ray, T. Cihlar, R. Jordan, M. R. Denison, R. S. Baric, Broad-spectrum antiviral GS-5734 inhibits both epidemic and zoonotic coronaviruses. *Sci. Transl. Med.* **9**, eaal3653 (2017). doi:10.1126/scitranslmed.aal3653 Medline

10. C. G. Rappazzo, L. V. Tse, C. I. Kaku, D. Wrapp, M. Sakharkar, D. Huang, L. M. Deveau, T. J. Yockachonis, A. S. Herbert, M. B. Battles, C. M. O’Brien, M. E. Brown, J. C. Geoghegan, J. Belk, L. Peng, L. Yang, Y. Hou, T. D. Scobey, D. R. Burton, D. Nemazee,

J. M. Dye, J. E. Voss, B. M. Gunn, J. S. McLellan, R. S. Baric, L. E. Gralinski, L. M. Walker, Broad and potent activity against SARS-like viruses by an engineered human monoclonal antibody. *Science* **371**, 823–829 (2021). doi:10.1126/science.abf4830 Medline

11. D. R. Martinez *et al.*, A broadly neutralizing antibody protects against SARS-CoV, pre-emergent bat CoVs, and SARS-CoV-2 variants in mice. *bioRxiv* [Preprint] 28 April 2021. doi:10.1101/2021.04.27.441655.

12. W. N. Voss, Y. J. Hou, N. V. Johnson, G. Delidakis, J. E. Kim, K. Javanmardi, A. P. Horton, F. Bartzoka, C. J. Paresi, Y. Tanno, C.-W. Chou, S. A. Abbasi, W. Pickens, K. George, D. R. Boutz, D. M. Towers, J. R. McDaniel, D. Billick, J. Goike, L. Rowe, D. Batra, J. Pohl, J. Lee, S. Gangappa, S. Sambhara, M. Gadush, N. Wang, M. D. Person, B. L. Iverson, J. D. Gollihar, J. M. Dye, A. S. Herbert, I. J. Finkelstein, R. S. Baric, J. S. McLellan, G. Georgiou, J. J. Lavinder, G. C. Ippolito, Prevalent, protective, and convergent IgG recognition of SARS-CoV-2 non-RBD spike epitopes. *Science* **372**, 1108–1112 (2021). doi:10.1126/science.abg5268 Medline

13. C. Graham, J. Seow, I. Huettner, H. Khan, N. Kouphou, S. Acors, H. Winstone, S. Pickering, R. P. Galao, L. Dupont, M. J. Lista, J. M. Jimenez-Guardeño, A. G. Laing, Y. Wu, M. Joseph, L. Muir, M. J. van Gils, W. M. Ng, H. M. E. Duyvesteyn, Y. Zhao, T. A. Bowden, M. Shankar-Hari, A. Rosa, P. Cherepanov, L. E. McCoy, A. C. Hayday, S. J. D. Neil, M. H. Malim, K. J. Doores, Neutralization potency of monoclonal antibodies recognizing dominant and subdominant epitopes on SARS-CoV-2 Spike is impacted by the B.1.1.7 variant. *Immunity* **54**, 1276–1289.e6 (2021). doi:10.1016/j.immuni.2021.03.023 Medline

14. L. Premkumar, B. Segovia-Chumbez, R. Jadi, D. R. Martinez, R. Raut, A. Markmann, C. Cornaby, L. Bartelt, S. Weiss, Y. Park, C. E. Edwards, E. Weimer, E. M. Scherer, N. Rouphael, S. Edupuganti, D. Weiskopf, L. V. Tse, Y. J. Hou, D. Margolis, A. Sette, M. H. Collins, J. Schmitz, R. S. Baric, A. M. de Silva, The receptor binding domain of the viral spike protein is an immunodominant and highly specific target of antibodies in SARS-CoV-2 patients. *Sci. Immunol.* **5**, eabc8413 (2020). doi:10.1126/sciimmunol.abc8413 Medline

15. L. Liu, P. Wang, M. S. Nair, J. Yu, M. Rapp, Q. Wang, Y. Luo, J. F.-W. Chan, V. Sahi, A. Figueroa, X. V. Guo, G. Cerutti, J. Bimela, J. Gorman, T. Zhou, Z. Chen, K.-Y. Yuen, P. D. Kwong, J. G. Sodroski, M. T. Yin, Z. Sheng, Y. Huang, L. Shapiro, D. D. Ho, Potent neutralizing antibodies against multiple epitopes on SARS-CoV-2 spike. *Nature* **584**, 450–456 (2020). doi:10.1038/s41586-020-2571-7 Medline

16. L. Dai, T. Zheng, K. Xu, Y. Han, L. Xu, E. Huang, Y. An, Y. Cheng, S. Li, M. Liu, M. Yang, Y. Li, H. Cheng, Y. Yuan, W. Zhang, C. Ke, G. Wong, J. Qi, C. Qin, J. Yan, G. F. Gao, A Universal Design of Betacoronavirus Vaccines against COVID-19, MERS, and SARS. *Cell* **182**, 722–733.e11 (2020). doi:10.1016/j.cell.2020.06.035 Medline

17. D. Li *et al.*, The functions of SARS-CoV-2 neutralizing and infection-enhancing antibodies in vitro and in mice and nonhuman primates. *bioRxiv* [Preprint] 2 January 2021. doi:10.1101/2020.12.31.424729.

18. A. R. Shiakolas, K. J. Kramer, D. Wrapp, S. I. Richardson, A. Schäfer, S. Wall, N. Wang, K. Janowska, K. A. Pilewski, R. Venkat, R. Parks, N. P. Manamela, N. Raju, E. F. Fechter, C. M. Holt, N. Suryadevara, R. E. Chen, D. R. Martinez, R. S. Nargi, R. E. Sutton, J. E. Ledgerwood, B. S. Graham, M. S. Diamond, B. F. Haynes, P. Acharya, R. H. Carnahan, J. E. Crowe Jr., R. S. Baric, L. Morris, J. S. McLellan, I. S. Georgiev, Cross-reactive coronavirus antibodies with diverse epitope specificities and Fc effector functions. *Cell Rep. Med.* **2**, 100313 (2021). doi:10.1016/j.xcrm.2021.100313 Medline

19. M. McCallum, A. De Marco, F. A. Lempp, M. A. Tortorici, D. Pinto, A. C. Walls, M. Beltramello, A. Chen, Z. Liu, F. Zatta, S. Zepeda, J. di Iulio, J. E. Bowen, M. Montiel-Ruiz, J. Zhou, L. E. Rosen, S. Bianchi, B. Guarino, C. S. Fregni, R. Abdelnabi, S. C. Foo, P. W. Rothlauf, L.-M. Bloyet, F. Benigni, E. Cameroni, J. Neyts, A. Riva, G. Snell, A. Telenti, S. P. J. Whelan, H. W. Virgin, D. Corti, M. S. Pizzuto, D. Veesler, N-terminal domain antigenic mapping reveals a site of vulnerability for SARS-CoV-2. *Cell* **184**, 2332–2347.e16 (2021). doi:10.1016/j.cell.2021.03.028 Medline

20. N. Suryadevara, S. Shrihari, P. Gilchuk, L. A. VanBlargan, E. Binshtein, S. J. Zost, R. S. Nargi, R. E. Sutton, E. S. Winkler, E. C. Chen, M. E. Fouch, E. Davidson, B. J. Doranz, R. E. Chen, P.-Y. Shi, R. H. Carnahan, L. B. Thackray, M. S. Diamond, J. E. Crowe Jr., Neutralizing and protective human monoclonal antibodies recognizing the N-terminal domain of the SARS-CoV-2 spike protein. *Cell* **184**, 2316–2331.e15 (2021). doi:10.1016/j.cell.2021.03.029 Medline

21. M. M. Becker, R. L. Graham, E. F. Donaldson, B. Rockx, A. C. Sims, T. Sheahan, R. J. Pickles, D. Corti, R. E. Johnston, R. S. Baric, M. R. Denison, Synthetic recombinant bat SARS-like coronavirus is infectious in cultured cells and in mice. *Proc. Natl. Acad. Sci. U.S.A.* **105**, 19944–19949 (2008). doi:10.1073/pnas.0808116105 Medline

22. D. Laczkó, M. J. Hogan, S. A. Toulmin, P. Hicks, K. Lederer, B. T. Gaudette, D. Castaño, F. Amanat, H. Muramatsu, T. H. Oguin 3rd, A. Ojha, L. Zhang, Z. Mu, R. Parks, T. B. Manzoni, B. Roper, S. Strohmeier, I. Tombácz, L. Arwood, R. Nachbagauer, K. Karikó, J. Greenhouse, L. Pessaint, M. Porto, T. Putman-Taylor, A. Strasbaugh, T.-A. Campbell, P. J. C. Lin, Y. K. Tam, G. D. Sempowski, M. Farzan, H. Choe, K. O. Saunders, B. F. Haynes, H. Andersen, L. C. Eisenlohr, D. Weissman, F. Krammer, P. Bates, D. Allman, M. Locci, N. Pardi, A Single Immunization with Nucleoside-Modified mRNA Vaccines Elicits Strong Cellular and Humoral Immune Responses against SARS-CoV-2 in Mice. *Immunity* **53**, 724–732.e7 (2020). doi:10.1016/j.immuni.2020.07.019 Medline

23. P. Zhou *et al.*, A protective broadly cross-reactive human antibody defines a conserved site of vulnerability on beta-coronavirus spikes. *bioRxiv* [Preprint] 31 March 2021. doi:10.1101/2021.03.30.437769.

24. K. Lederer, D. Castaño, D. Gómez Atria, T. H. Oguin 3rd, S. Wang, T. B. Manzoni, H. Muramatsu, M. J. Hogan, F. Amanat, P. Cherubin, K. A. Lundgreen, Y. K. Tam, S. H. Y. Fan, L. C. Eisenlohr, I. Maillard, D. Weissman, P. Bates, F. Krammer, G. D. Sempowski, N. Pardi, M. Locci, SARS-CoV-2 mRNA Vaccines Foster Potent Antigen-Specific Germinal Center Responses Associated with Neutralizing Antibody Generation. *Immunity* **53**, 1281–1295.e5 (2020). doi:10.1016/j.immuni.2020.11.009 Medline

25. D. E. Gordon, J. Hiatt, M. Bouhaddou, V. V. Rezelj, S. Ulferts, H. Braberg, A. S. Jureka, K. Obernier, J. Z. Guo, J. Batra, R. M. Kaake, A. R. Weckstein, T. W. Owens, M. Gupta, S. Pourmal, E. W. Titus, M. Cakir, M. Soucheray, M. McGregor, Z. Cakir, G. Jang, M. J. O'Meara, T. A. Tummino, Z. Zhang, H. Foussard, A. Rojc, Y. Zhou, D. Kuchenov, R. Hüttenhain, J. Xu, M. Eckhardt, D. L. Swaney, J. M. Fabius, M. Ummadi, B. Tutuncuoglu, U. Rathore, M. Modak, P. Haas, K. M. Haas, Z. Z. C. Naing, E. H. Pulido, Y. Shi, I. Barrio-Hernandez, D. Memon, E. Petsalaki, A. Dunham, M. C. Marrero, D. Burke, C. Koh, T. Vallet, J. A. Silvas, C. M. Azumaya, C. Billesbølle, A. F. Brilot, M. G. Campbell, A. Diallo, M. S. Dickinson, D. Diwanji, N. Herrera, N. Hoppe, H. T. Kratochvil, Y. Liu, G. E. Merz, M. Moritz, H. C. Nguyen, C. Nowotny, C. Puchades, A. N. Rizo, U. Schulze-Gahmen, A. M. Smith, M. Sun, I. D. Young, J. Zhao, D. Asarnow, J. Biel, A. Bowen, J. R. Braxton, J. Chen, C. M. Chio, U. S. Chio, I. Deshpande, L. Doan, B. Faust, S. Flores, M. Jin, K. Kim, V. L. Lam, F. Li, J. Li, Y.-L. Li, Y. Li, X. Liu, M. Lo, K. E. Lopez, A. A. Melo, F. R. Moss 3rd, P. Nguyen, J. Paulino, K. I. Pawar, J. K. Peters, T. H. Pospiech Jr., M. Safari, S. Sangwan, K. Schaefer, P. V. Thomas, A. C. Thwin, R. Trenker, E. Tse, T. K. M. Tsui, F. Wang, N. Whitis, Z. Yu, K. Zhang, Y. Zhang, F. Zhou, D. Saltzberg, A. J. Hodder, A. S. Shun-Shion, D. M. Williams, K. M. White, R. Rosales, T. Kehrer, L. Miorin, E. Moreno, A. H. Patel, S. Rihn, M. M. Khalid, A. Vallejo-Gracia, P. Fozouni, C. R. Simoneau, T. L. Roth, D. Wu, M. A. Karim, M. Ghoussaini, I. Dunham, F. Berardi, S. Weigang, M. Chazal, J. Park, J. Logue, M. McGrath, S. Weston, R. Haupt, C. J. Hastie, M. Elliott, F. Brown, K. A. Burness, E. Reid, M. Dorward, C. Johnson, S. G. Wilkinson, A. Geyer, D. M. Giesel, C. Baillie, S. Raggett, H. Leech, R. Toth, N. Goodman, K. C. Keough, A. L. Lind, R. J. Klesh, K. R. Hemphill, J. Carlson-Stevermer, J. Oki, K. Holden, T. Maures, K. S. Pollard, A. Sali, D. A. Agard, Y. Cheng, J. S. Fraser, A. Frost, N. Jura, T. Kortemme, A. Manglik, D. R. Southworth, R. M. Stroud, D. R. Alessi, P. Davies, M. B. Frieman, T. Ideker, C. Abate, N. Jouvenet, G. Kochs, B. Shoichet, M. Ott, M. Palmarini, K. M. Shokat, A. García-Sastre, J. A. Rassen, R. Grosse, O. S. Rosenberg, K. A. Verba, C. F. Basler, M. Vignuzzi, A. A. Peden, P. Beltrao, N. J. Krogan; QCRG Structural Biology Consortium; Zoonomia Consortium, Comparative host-coronavirus protein interaction networks reveal pan-viral disease mechanisms. *Science* **370**, eabe9403 (2020). doi:10.1126/science.abe9403 Medline

26. J. Y. Li, C.-H. Liao, Q. Wang, Y.-J. Tan, R. Luo, Y. Qiu, X.-Y. Ge, The ORF6, ORF8 and nucleocapsid proteins of SARS-CoV-2 inhibit type I interferon signaling pathway. *Virus Res.* **286**, 198074 (2020). doi:10.1016/j.virusres.2020.198074 Medline

27. Y. C. F. Su, D. E. Anderson, B. E. Young, M. Linster, F. Zhu, J. Jayakumar, Y. Zhuang, S. Kalimuddin, J. G. H. Low, C. W. Tan, W. N. Chia, T. M. Mak, S. Octavia, J. M. Chavatte, R. T. C. Lee, S. Pada, S. Y. Tan, L. Sun, G. Z. Yan, S. Maurer-Stroh, I. H. Mendenhall, Y. S. Leo, D. C. Lye, L. F. Wang, G. J. D. Smith, Discovery and Genomic Characterization of a 382-Nucleotide Deletion in ORF7b and ORF8 during the Early Evolution of SARS-CoV-2. *mBio* **11**, e01610-20 (2020). 10.1128/mBio.01610-20 Medline

28. B. E. Young, S.-W. Fong, Y.-H. Chan, T.-M. Mak, L. W. Ang, D. E. Anderson, C. Y.-P. Lee, S. N. Amrun, B. Lee, Y. S. Goh, Y. C. F. Su, W. E. Wei, S. Kalimuddin, L. Y. A. Chai, S. Pada, S. Y. Tan, L. Sun, P. Parthasarathy, Y. Y. C. Chen, T. Barkham, R. T. P. Lin, S.

Maurer-Stroh, Y.-S. Leo, L.-F. Wang, L. Renia, V. J. Lee, G. J. D. Smith, D. C. Lye, L. F. P. Ng, Effects of a major deletion in the SARS-CoV-2 genome on the severity of infection and the inflammatory response: An observational cohort study. *Lancet* **396**, 603–611 (2020). doi:10.1016/S0140-6736(20)31757-8 Medline

29. T. P. Sheahan, A. C. Sims, S. R. Leist, A. Schäfer, J. Won, A. J. Brown, S. A. Montgomery, A. Hogg, D. Babusis, M. O. Clarke, J. E. Spahn, L. Bauer, S. Sellers, D. Porter, J. Y. Feng, T. Cihlar, R. Jordan, M. R. Denison, R. S. Baric, Comparative therapeutic efficacy of remdesivir and combination lopinavir, ritonavir, and interferon beta against MERS-CoV. *Nat. Commun.* **11**, 222 (2020). doi:10.1038/s41467-019-13940-6 Medline

30. M. E. Schmidt, C. J. Knudson, S. M. Hartwig, L. L. Pewe, D. K. Meyerholz, R. A. Langlois, J. T. Harty, S. M. Varga, Memory CD8 T cells mediate severe immunopathology following respiratory syncytial virus infection. *PLOS Pathog.* **14**, e1006810 (2018). doi:10.1371/journal.ppat.1006810 Medline

31. M. Bolles, D. Deming, K. Long, S. Agnihothram, A. Whitmore, M. Ferris, W. Funkhouser, L. Gralinski, A. Totura, M. Heise, R. S. Baric, A double-inactivated severe acute respiratory syndrome coronavirus vaccine provides incomplete protection in mice and induces increased eosinophilic proinflammatory pulmonary response upon challenge. *J. Virol.* **85**, 12201–12215 (2011). doi:10.1128/JVI.06048-11 Medline

32. L. A. Jackson, E. J. Anderson, N. G. Rouphael, P. C. Roberts, M. Makhene, R. N. Coler, M. P. McCullough, J. D. Chappell, M. R. Denison, L. J. Stevens, A. J. Pruijssers, A. McDermott, B. Flach, N. A. Doria-Rose, K. S. Corbett, K. M. Morabito, S. O’Dell, S. D. Schmidt, P. A. Swanson 2nd, M. Padilla, J. R. Mascola, K. M. Neuzil, H. Bennett, W. Sun, E. Peters, M. Makowski, J. Albert, K. Cross, W. Buchanan, R. Pikaart-Tautges, J. E. Ledgerwood, B. S. Graham, J. H. Beigel; mRNA-1273 Study Group, An mRNA Vaccine against SARS-CoV-2 - Preliminary Report. *N. Engl. J. Med.* **383**, 1920–1931 (2020). doi:10.1056/NEJMoa2022483 Medline

33. E. E. Walsh, R. W. Frenck Jr., A. R. Falsey, N. Kitchin, J. Absalon, A. Gurtman, S. Lockhart, K. Neuzil, M. J. Mulligan, R. Bailey, K. A. Swanson, P. Li, K. Koury, W. Kalina, D. Cooper, C. Fontes-Garfias, P.-Y. Shi, Ö. Türeci, K. R. Tompkins, K. E. Lyke, V. Raabe, P. R. Dormitzer, K. U. Jansen, U. Şahin, W. C. Gruber, Safety and Immunogenicity of Two RNA-Based Covid-19 Vaccine Candidates. *N. Engl. J. Med.* **383**, 2439–2450 (2020). doi:10.1056/NEJMoa2027906 Medline

34. L. R. Baden, H. M. El Sahly, B. Essink, K. Kotloff, S. Frey, R. Novak, D. Diemert, S. A. Spector, N. Rouphael, C. B. Creech, J. McGettigan, S. Khetan, N. Segall, J. Solis, A. Brosz, C. Fierro, H. Schwartz, K. Neuzil, L. Corey, P. Gilbert, H. Janes, D. Follmann, M. Marovich, J. Mascola, L. Polakowski, J. Ledgerwood, B. S. Graham, H. Bennett, R. Pajon, C. Knightly, B. Leav, W. Deng, H. Zhou, S. Han, M. Ivarsson, J. Miller, T. Zaks; COVE Study Group, Efficacy and Safety of the mRNA-1273 SARS-CoV-2 Vaccine. *N. Engl. J. Med.* **384**, 403–416 (2021). doi:10.1056/NEJMoa2035389 Medline

35. K. Wu, A. P. Werner, M. Koch, A. Choi, E. Narayanan, G. B. E. Stewart-Jones, T. Colpitts, H. Bennett, S. Boyoglu-Barnum, W. Shi, J. I. Moliva, N. J. Sullivan, B. S. Graham, A. Carfi, K. S. Corbett, R. A. Seder, D. K. Edwards, Serum Neutralizing Activity Elicited by

mRNA-1273 Vaccine. *N. Engl. J. Med.* **384**, 1468–1470 (2021). doi:10.1056/NEJMc2102179 Medline

36. M. M. Sauer, M. A. Tortorici, Y.-J. Park, A. C. Walls, L. Homad, O. J. Acton, J. E. Bowen, C. Wang, X. Xiong, W. de van der Schueren, J. Quispe, B. G. Hoffstrom, B.-J. Bosch, A. T. McGuire, D. Veesler, Structural basis for broad coronavirus neutralization. *Nat. Struct. Mol. Biol.* **28**, 478–486 (2021). doi:10.1038/s41594-021-00596-4 Medline

37. A. A. Cohen, P. N. P. Gnanapragasam, Y. E. Lee, P. R. Hoffman, S. Ou, L. M. Kakutani, J. R. Keeffe, H.-J. Wu, M. Howarth, A. P. West, C. O. Barnes, M. C. Nussenzweig, P. J. Bjorkman, Mosaic nanoparticles elicit cross-reactive immune responses to zoonotic coronaviruses in mice. *Science* **371**, 735–741 (2021). doi:10.1126/science.abf6840 Medline

38. K. O. Saunders, E. Lee, R. Parks, D. R. Martinez, D. Li, H. Chen, R. J. Edwards, S. Gobeil, M. Barr, K. Mansouri, S. M. Alam, L. L. Sutherland, F. Cai, A. M. Sanzone, M. Berry, K. Manne, K. W. Bock, M. Minai, B. M. Nagata, A. B. Kapingidza, M. Azoitei, L. V. Tse, T. D. Scobey, R. L. Spreng, R. W. Rountree, C. T. DeMarco, T. N. Denny, C. W. Woods, E. W. Petzold, J. Tang, T. H. Oguin 3rd, G. D. Sempowski, M. Gagne, D. C. Douek, M. A. Tomai, C. B. Fox, R. Seder, K. Wiehe, D. Weissman, N. Pardi, H. Golding, S. Khurana, P. Acharya, H. Andersen, M. G. Lewis, I. N. Moore, D. C. Montefiori, R. S. Baric, B. F. Haynes, Neutralizing antibody vaccine for pandemic and pre-emergent coronaviruses. *Nature* (2021). doi:10.1038/s41586-021-03594-0 Medline

39. L. R. Banner, J. G. Keck, M. M. Lai, A clustering of RNA recombination sites adjacent to a hypervariable region of the peplomer gene of murine coronavirus. *Virology* **175**, 548–555 (1990). doi:10.1016/0042-6822(90)90439-X Medline

40. J. G. Keck, L. H. Soe, S. Makino, S. A. Stohlman, M. M. Lai, RNA recombination of murine coronaviruses: Recombination between fusion-positive mouse hepatitis virus A59 and fusion-negative mouse hepatitis virus 2. *J. Virol.* **62**, 1989–1998 (1988). doi:10.1128/jvi.62.6.1989-1998.1988 Medline

41. A. W. Freyn, J. Ramos da Silva, V. C. Rosado, C. M. Bliss, M. Pine, B. L. Mui, Y. K. Tam, T. D. Madden, L. C. de Souza Ferreira, D. Weissman, F. Krammer, L. Coughlan, P. Palese, N. Pardi, R. Nachbagauer, A Multi-Targeting, Nucleoside-Modified mRNA Influenza Virus Vaccine Provides Broad Protection in Mice. *Mol. Ther.* **28**, 1569–1584 (2020). doi:10.1016/j.ymthe.2020.04.018 Medline

42. S. R. Leist, K. H. Dinnon 3rd, A. Schäfer, L. V. Tse, K. Okuda, Y. J. Hou, A. West, C. E. Edwards, W. Sanders, E. J. Fritch, K. L. Gully, T. Scobey, A. J. Brown, T. P. Sheahan, N. J. Moorman, R. C. Boucher, L. E. Gralinski, S. A. Montgomery, R. S. Baric, A Mouse-Adapted SARS-CoV-2 Induces Acute Lung Injury and Mortality in Standard Laboratory Mice. *Cell* **183**, 1070–1085.e12 (2020). doi:10.1016/j.cell.2020.09.050 Medline

43. A. Roberts, D. Deming, C. D. Paddock, A. Cheng, B. Yount, L. Vogel, B. D. Herman, T. Sheahan, M. Heise, G. L. Genrich, S. R. Zaki, R. Baric, K. Subbarao, A mouse-adapted SARS-coronavirus causes disease and mortality in BALB/c mice. *PLOS Pathog.* **3**, e5 (2007). doi:10.1371/journal.ppat.0030005 Medline
