# Supplementary material for: Chimeric spike mRNA vaccines protect against Sarbecovirus challenge in mice
Source: Science. 2021 Aug 27;373(6558):991–8. doi: 10.1126/science.abi4506 (PMC8899822; doi:10.1126/science.abi4506)
Supplement: Supplementary file 2 — MDAR Reproducibility Checklist [file science.abi4506_reproducibility-checklist.pdf]

Materials Design Analysis Reporting (MDAR)
Checklist for Authors

The MDAR framework establishes a minimum set of requirements in transparent reporting applicable to studies in the life sciences (see Statement of Task: doi:10.31222/osf.io/9sm4x.). The MDAR checklist is a tool for authors, editors, and others seeking to adopt the MDAR framework for transparent reporting in manuscripts and other outputs. Please refer to the MDAR Elaboration Document for additional context for the MDAR framework.

For all that apply, please note where in the manuscript the required information is provided.

Materials:

Newly created materials	indicate where provided: page no/section/legend)	n/a
The manuscript includes a dedicated "materials availability statement" providing transparent disclosure about availability of newly created materials including details on how materials can be accessed and describing any restrictions on access.	Please see Data and materials availability section	

Antibodies	indicate where provided: page no/section/legend)	n/a
For commercial reagents, provide supplier name, catalogue number and RRID, if available.	HRP conjugated goat anti-mouse IgG secondary antibody (SouthernBiotech 1030-05)<br><br>Strep-Tactin HRP, (IBA GmbH, Cat# 2-1502-001)<br><br>Eosinophil Peroxidase antibody (PA5-62200, Invitrogen)	

DNA and RNA sequences	indicate where provided: page no/section/legend)	n/a
**Short novel DNA or RNA including primers, probes:** Sequences should be included or deposited in a public repository.	n/a	x

Cell materials	indicate where provided: page no/section/legend	n/a
**Cell lines:** Provide species information, strain. Provide accession number in repository **OR** supplier name, catalog number, clone number, **OR** RRID.	Vero E6 ATCC (C1108)	
**Primary cultures:** Provide species, strain, sex of origin, genetic modification status.	n/a	x

Experimental animals	indicate where provided: page no/section/legend)	n/a
**Laboratory animals or Model organisms:** Provide species, strain, sex, age, genetic modification status. Provide accession number in repository **OR** supplier name, catalog number, clone number, **OR** RRID.	11-month old female BALB/c mice (Envigo - cat# 047)<br>15 week old Hfh4-ACE2 transgenic male mice - bred at UNC Chapel Hill	
**Animal observed in or captured from the field:** Provide species, sex, and age where possible.	n/a	x

Plants and microbes	indicate where provided: page no/section/legend)	n/a

**Plants:** provide species and strain, ecotype and cultivar where relevant, unique accession number if available, and source (including location for collected wild specimens).	n/a	x
**Microbes:** provide species and strain, unique accession number if available, and source.	viruses for live virus neutralization assays<br><br>SARS-CoV-2 full length virus - accession #: MN908947<br>SARS-CoV full length virus - accession #: AY278741<br>WIV-1 full length virus - accession #: KF367457.1<br>RsSHC014 full length virus - accession #:KC881005.1	

**Human research participants**	**indicate where provided: page no/section/legend) or state if these demographics were not collected**	**n/a**
If collected and within the bounds of privacy constraints report on age, sex and gender or ethnicity for all study participants.	n/a	x

Design:

**Study protocol**	**indicate where provided: page no/section/legend)**	**n/a**
If study protocol has been pre-registered, provide DOI. For clinical trials, provide the trial registration number **OR** cite DOI.	n/a	x

**Laboratory protocol**	**indicate where provided: page no/section/legend)**	**n/a**
Provide DOI **OR** other citation details if detailed step-by-step protocols are available.	n/a	x

**Experimental study design (statistics details)**		
**For in vivo studies:** State whether and how the following have been done	**indicate where provided: page no/section/legend. If it could have been done, but was not, write not done**	**n/a**
Sample size determination	For mouse experiments 10 mice per group per challenge virus were used to obtain power in statistical analyses	
Randomisation	n/a	x
Blinding	n/a	x
Inclusion/exclusion criteria	n/a	x

**Sample definition and in-laboratory replication**	**indicate where provided: page no/section/legend**	**n/a**
State number of times the experiment was replicated in laboratory.	All mouse vaccination and challenge studies were repeated twice independently to ensure reproducibility	
Define whether data describe technical or biological replicates.	ELISAs and neutralizations for sera from each animal at time points indicated in Fig. 2 Fig. 3 were performed in duplicate. Each value for LogAUC or ID50 represents the average of each duplicate.	

Ethics	indicate where provided: page no/section/legend	n/a
**Studies involving human participants:** State details of authority granting ethics approval (IRB or equivalent committee(s), provide reference number for approval.	n/a	x
**Studies involving experimental animals:** State details of authority granting ethics approval (IRB or equivalent committee(s), provide reference number for approval.	Animal procedures and experiments were performed in agreement with protocols approved by the IACUC protocol at UNC 20-199	x
**Studies involving specimen and field samples:** State if relevant permits obtained, provide details of authority approving study; if none were required, explain why.	n/a	x

Dual Use Research of Concern (DURC)	indicate where provided: page no/section/legend	n/a
If study is subject to dual use research of concern regulations, state the authority granting approval and reference number for the regulatory approval.	n/a	x

Analysis:

Attrition	indicate where provided: page no/section/legend	n/a
Describe whether exclusion criteria were preestablished. Report if sample or data points were omitted from analysis. If yes report if this was due to attrition or intentional exclusion and provide justification.	n/a	x

Statistics	indicate where provided: page no/section/legend	n/a
Describe statistical tests used and justify choice of tests.	Comparisons between mice measurements were done with a two-way ANOVA after Dunnett's multiple comparison correction. Mice lung and nasal titers were compared with a one-way ANOVA after a Tukey's multiple comparison correction. Comparisons between groups for ELISAs and neutralization assays were calculated with a Kruskal-Wallis test with a Dunnett's multiple comparison correction.	

Data availability	indicate where provided: page no/section/legend	n/a
For newly created and reused datasets, the manuscript includes a data availability statement that provides details for access or notes restrictions on access.	n/a	x
If newly created datasets are publicly available, provide accession number in repository **OR** DOI **OR** URL and licensing details where available.	n/a	x
If reused data is publicly available provide accession number in repository **OR** DOI **OR** URL, **OR** citation.	n/a	x

Code availability	indicate where provided: page no/section/legend	n/a
For all newly generated custom computer code/software/mathematical algorithm or re-used code essential for replicating the main findings of the study, the manuscript includes a data availability statement that provides details for access or notes restrictions.	n/a	X
If newly generated code is publicly available, provide accession number in repository, **OR** DOI **OR** URL and licensing details where available. State any	n/a	x

restrictions on code availability or accessibility.		
If reused code is publicly available provide accession number in repository **OR** DOI **OR** URL, **OR** citation.	n/a	x

Reporting

MDAR framework recommends adoption of discipline-specific guidelines, established and endorsed through community initiatives. Journals have their own policy about requiring specific guidelines and recommendations to complement MDAR.

Adherence to community standards	indicate where provided: page no/section/legend	n/a
State if relevant guidelines (e.g., ICMJE, MIBBI, ARRIVE) have been followed, and whether a checklist (e.g., CONSORT, PRISMA, ARRIVE) is provided with the manuscript.	n/a	
